# Supplementary figures and images for: Estimates of the number and distribution of zero-dose and under-immunised children across remote-rural, urban, and conflict-affected settings in low and middle-income countries
Source: PLOS Glob Public Health. 2022 Oct 26;2(10):e0001126. doi: 10.1371/journal.pgph.0001126 (PMC10021885; doi:10.1371/journal.pgph.0001126)

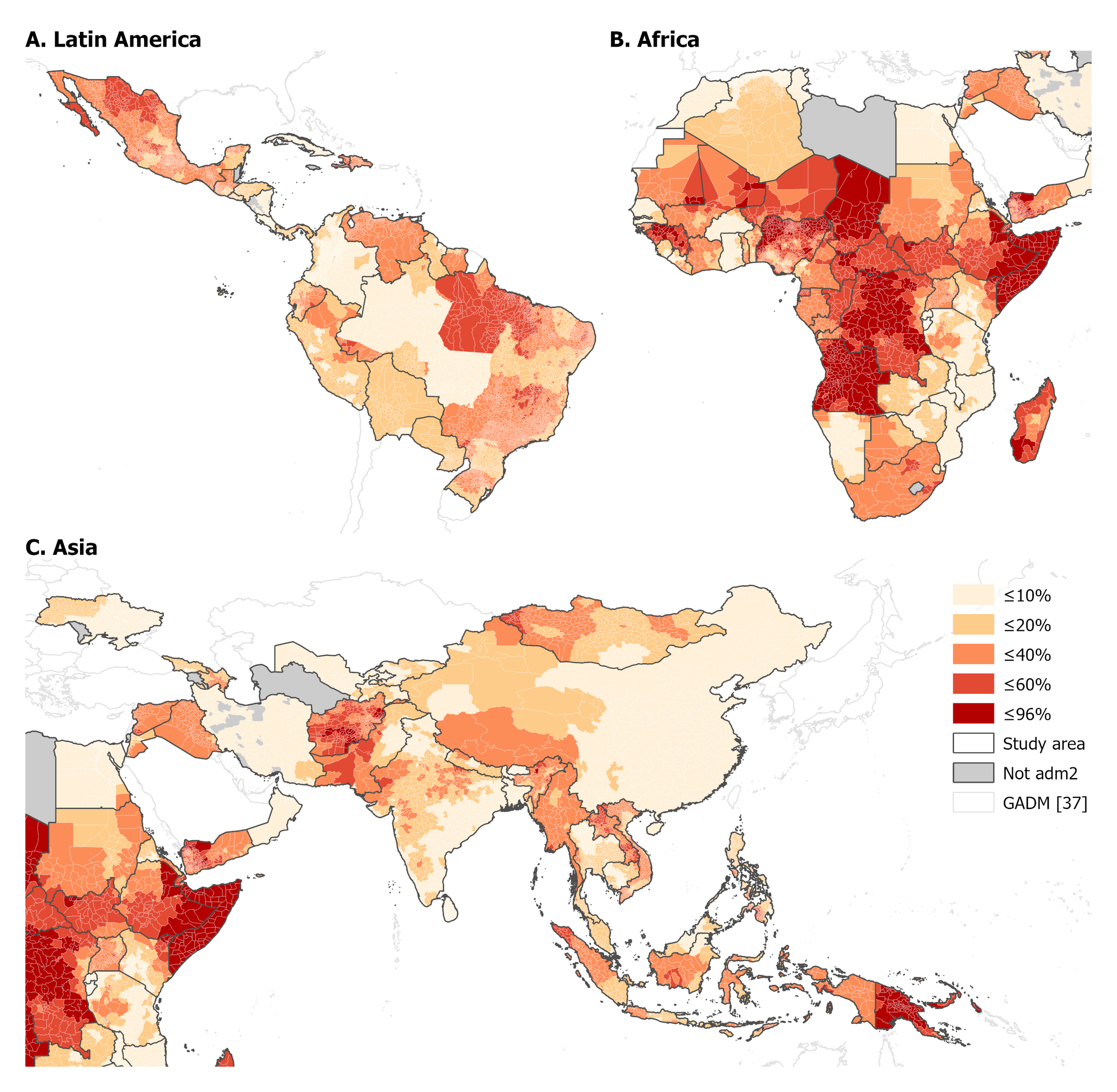

Supplement: S1 Fig — Proportion of children under 1 year of age estimated to have not received the third dose of the DTP vaccine in 2019 at administrative level 2 [31, 37] for Latin America (A), Africa (B), and Asia (C). (TIF) [file pgph.0001126.s001.tif]

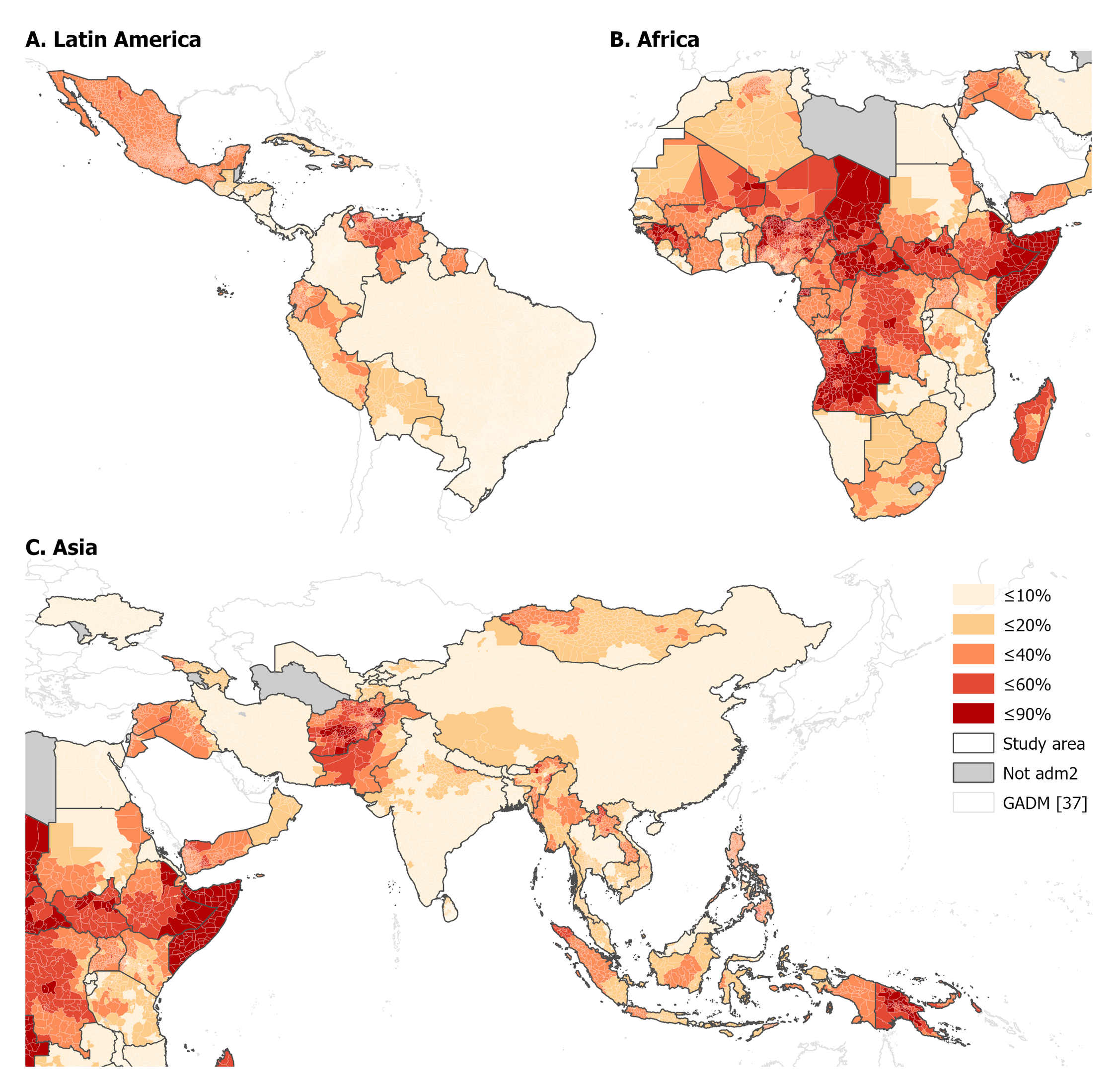

Supplement: S2 Fig — Proportion of children under 1 year of age estimated to have not received the first dose of the MCV vaccine in 2019 at administrative level 2 [31, 37] for Latin America (A), Africa (B), and Asia (C). (TIF) [file pgph.0001126.s002.tif]

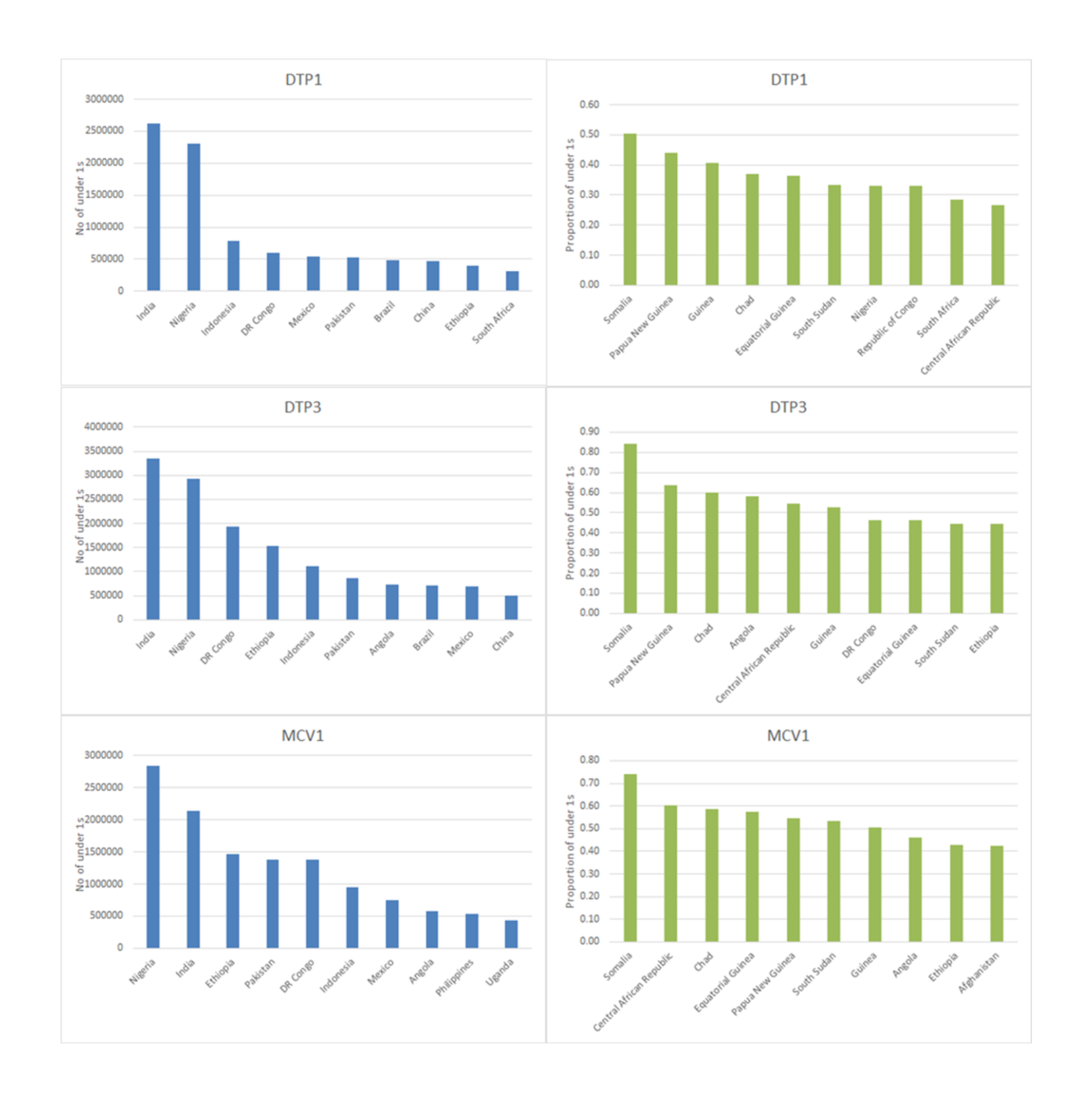

Supplement: S3 Fig — Graphs showing the top ten countries for children under 1 year of age not receiving DTP1/DTP3/MCV1 in 2019 measured by (left hand side) total number and (right hand side) proportion of the total children under 1 year of age; broken down by (a) first dose of DTP vaccine, (b) third dose of DTP vaccine; (c) first dose of MCV. (TIF) [file pgph.0001126.s003.tif]

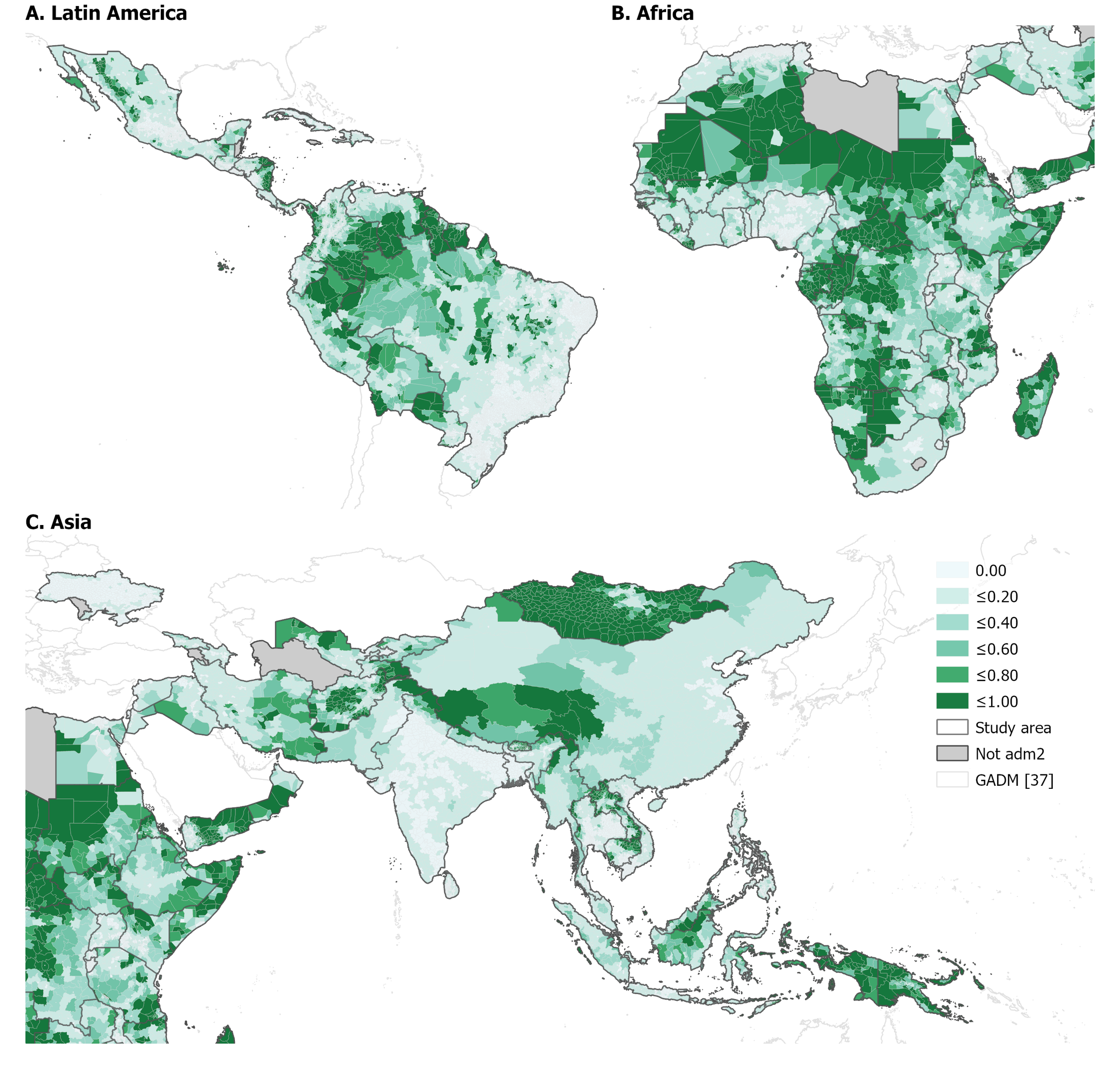

Supplement: S4 Fig — Proportion of children under 1 year of age in remote-rural areas estimated to have not received the third dose of the DTP vaccine in 2019 at administrative level 2 [31, 37] for Latin America (A), Africa (B), and Asia (C). (TIF) [file pgph.0001126.s004.tif]

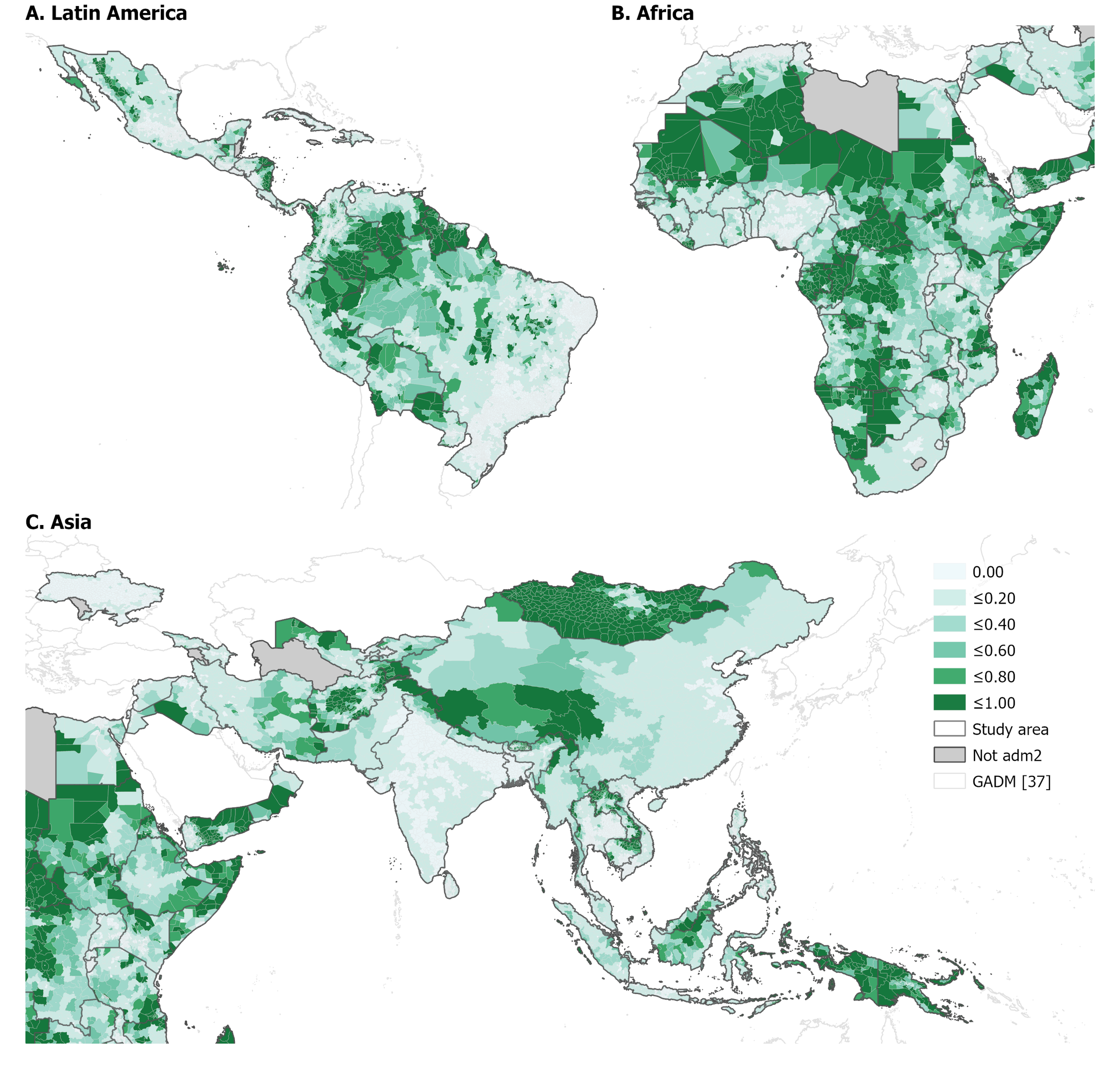

Supplement: S5 Fig — Proportion of children under 1 year of age in remote-rural areas estimated to have not received the first dose of the MCV vaccine in 2019 at administrative level 2 [31, 37] for Latin America (A), Africa (B), and Asia (C). (TIF) [file pgph.0001126.s005.tif]

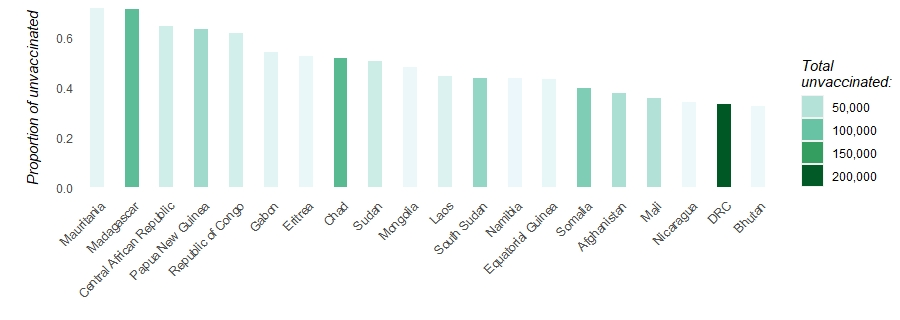

Supplement: S6 Fig — (TIF) [file pgph.0001126.s006.tif]

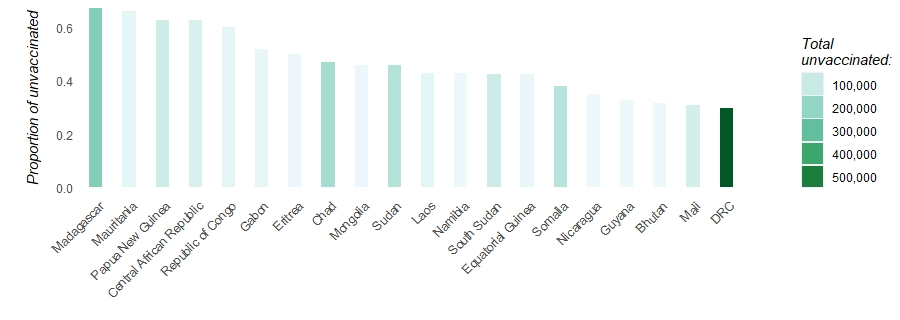

Supplement: S7 Fig — (TIF) [file pgph.0001126.s007.tif]

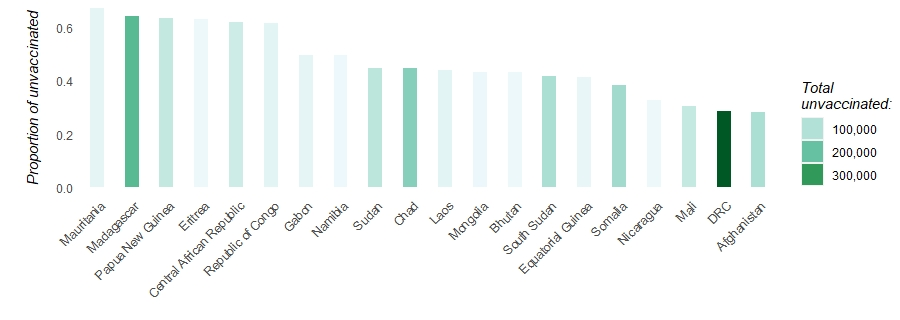

Supplement: S8 Fig — (TIF) [file pgph.0001126.s008.tif]

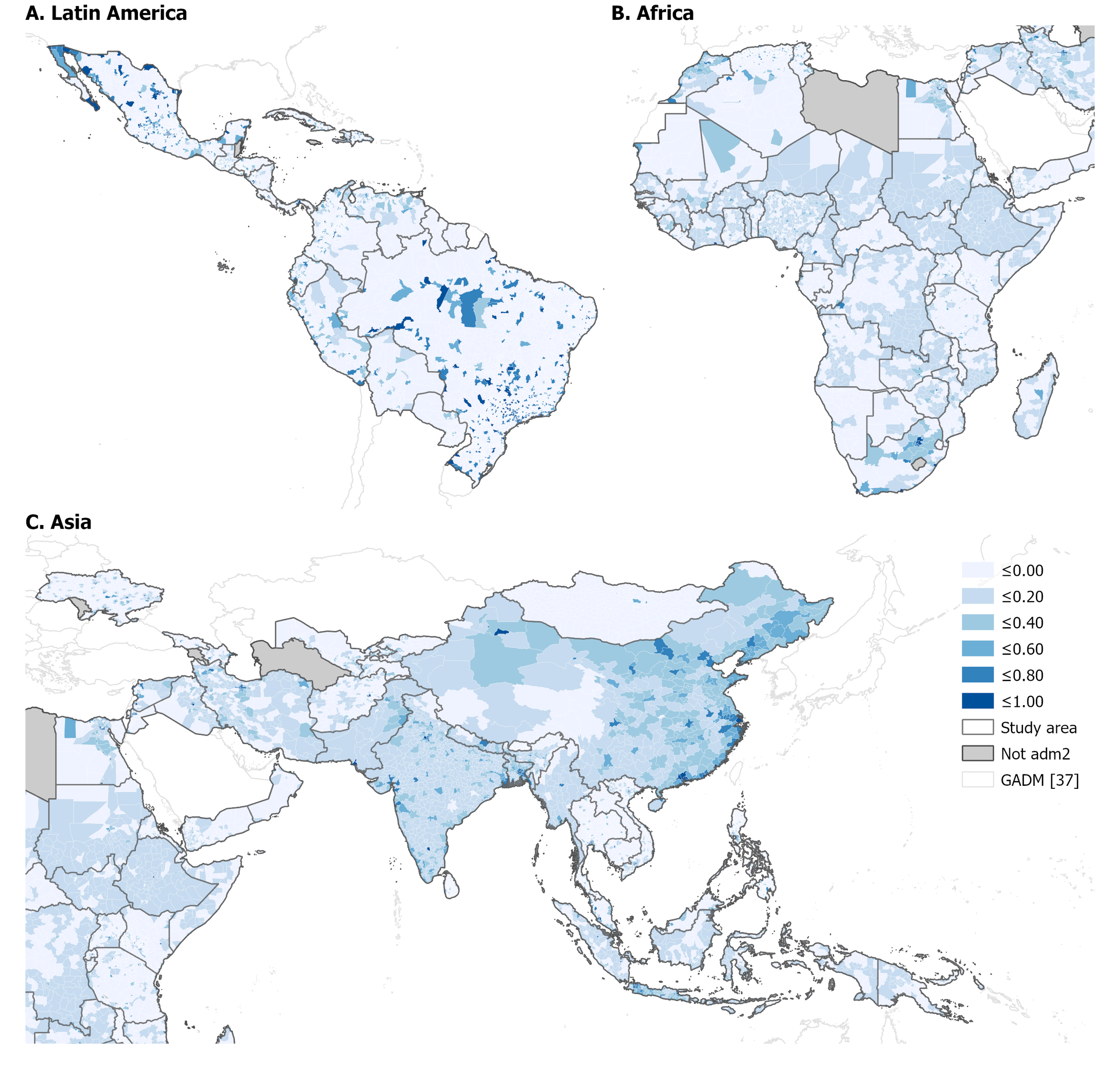

Supplement: S9 Fig — Proportion of children under 1 year of age in urban areas estimated to have not received the first dose of the DTP vaccine in 2019 at administrative level 2 [31, 37] for Latin America (A), Africa (B), and Asia (C). (TIF) [file pgph.0001126.s009.tif]

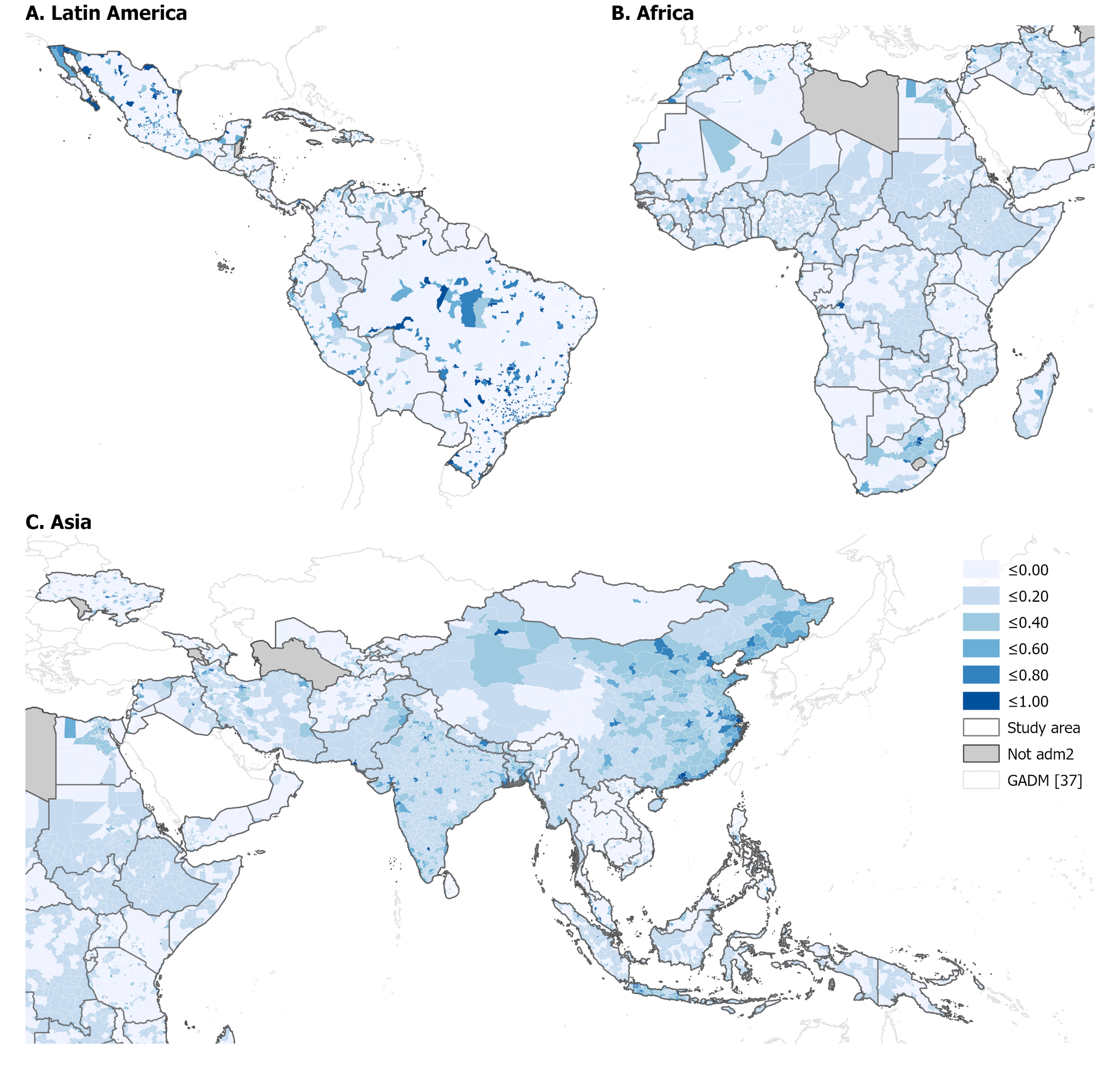

Supplement: S10 Fig — Proportion of children under 1 year of age in urban areas estimated to have not received the third dose of the DTP vaccine in 2019 at administrative level 2 [31, 37] for Latin America (A), Africa (B), and Asia (C). (TIF) [file pgph.0001126.s010.tif]

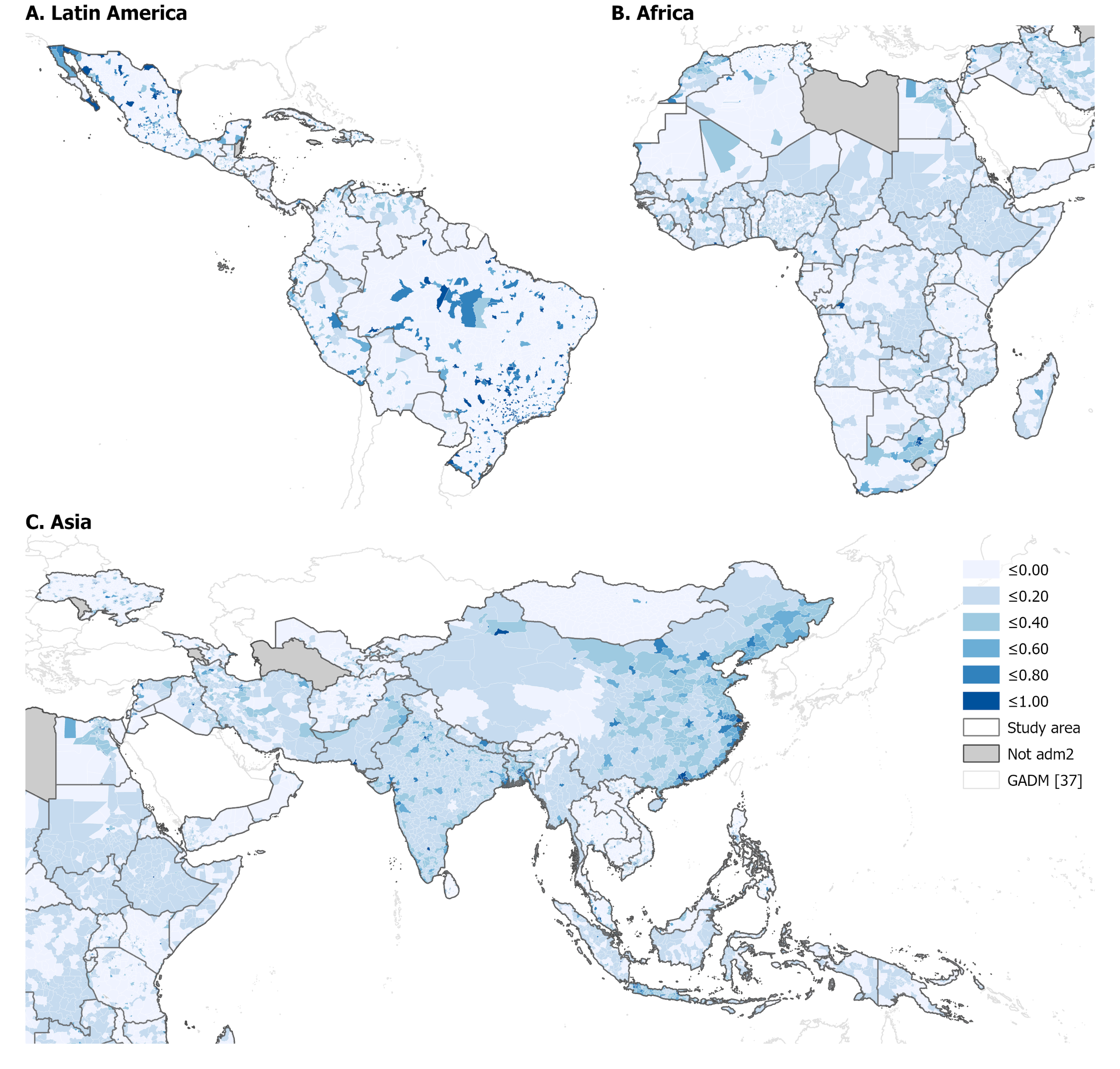

Supplement: S11 Fig — Proportion of children under 1 year of age in remote-rural areas estimated to have not received the first dose of the MCV vaccine in 2019 at administrative level 2 [31, 37] for Latin America (A), Africa (B), and Asia (C). (TIF) [file pgph.0001126.s011.tif]

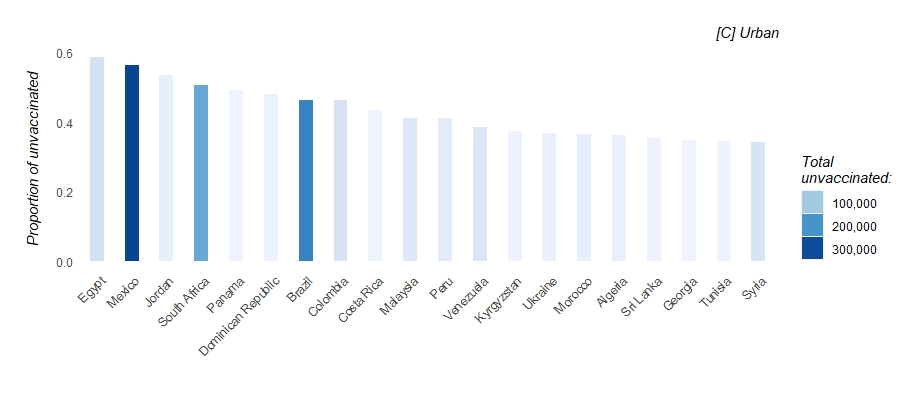

Supplement: S12 Fig — (TIF) [file pgph.0001126.s012.tif]

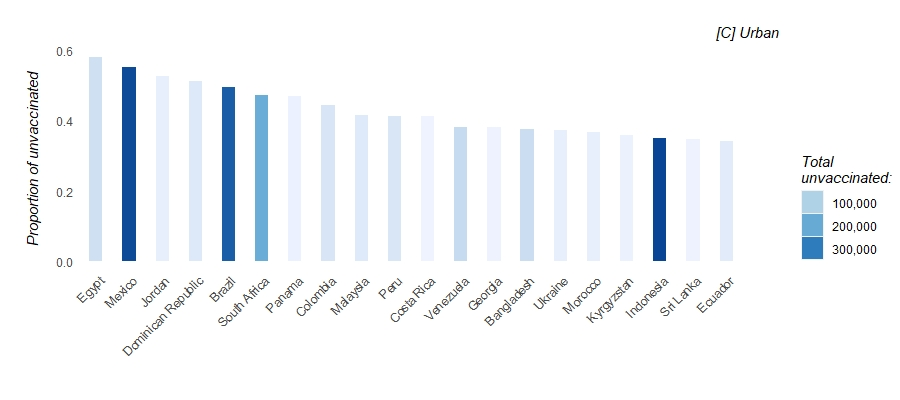

Supplement: S13 Fig — (TIF) [file pgph.0001126.s013.tif]

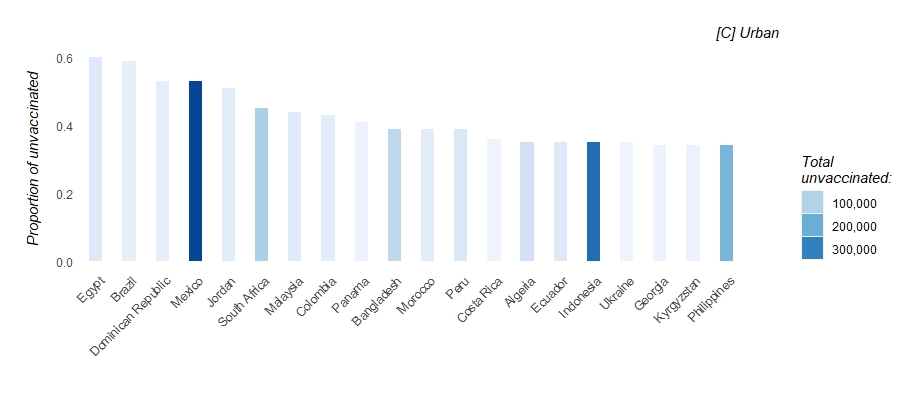

Supplement: S14 Fig — (TIF) [file pgph.0001126.s014.tif]

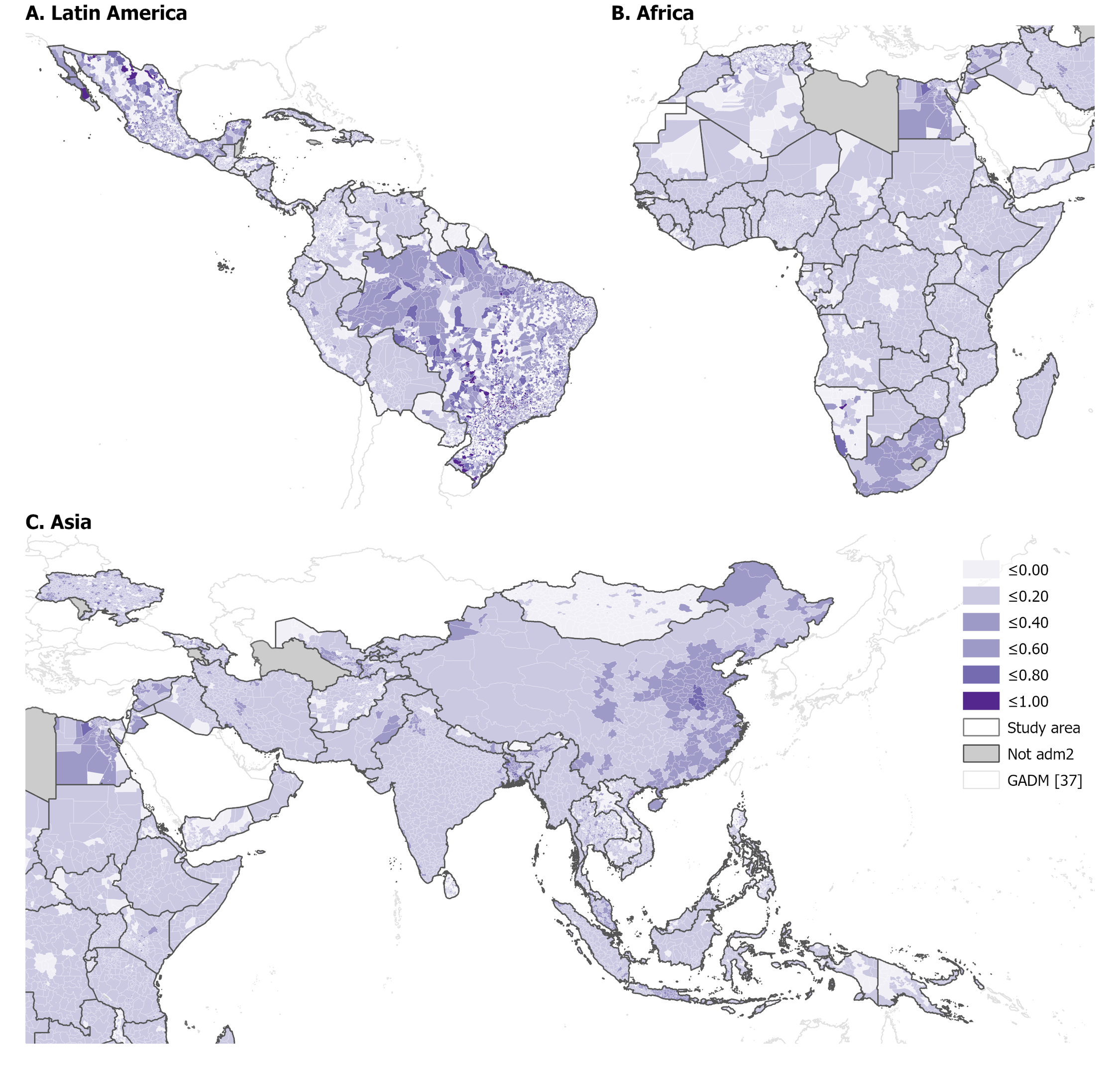

Supplement: S15 Fig — Proportion of children under 1 year of age in peri-urban areas estimated to have not received the first dose of the DTP vaccine in 2019 at administrative level 2 [31, 37] for Latin America (A), Africa (B), and Asia (C). (TIF) [file pgph.0001126.s015.tif]

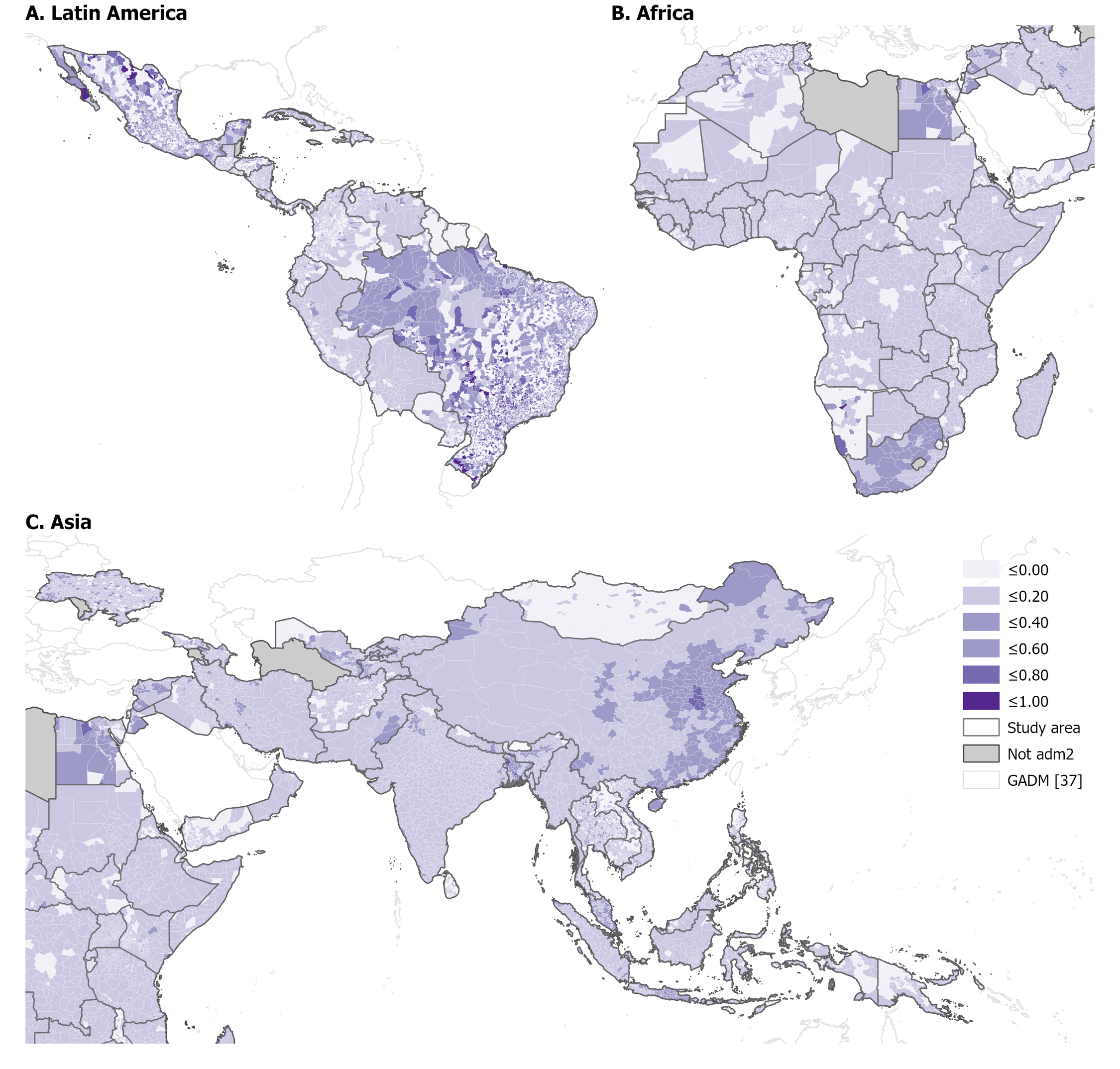

Supplement: S16 Fig — Proportion of children under 1 year of age in peri-urban areas estimated to have not received the third dose of the DTP vaccine in 2019 at administrative level 2 [31, 37] for Latin America (A), Africa (B), and Asia (C). (TIF) [file pgph.0001126.s016.tif]

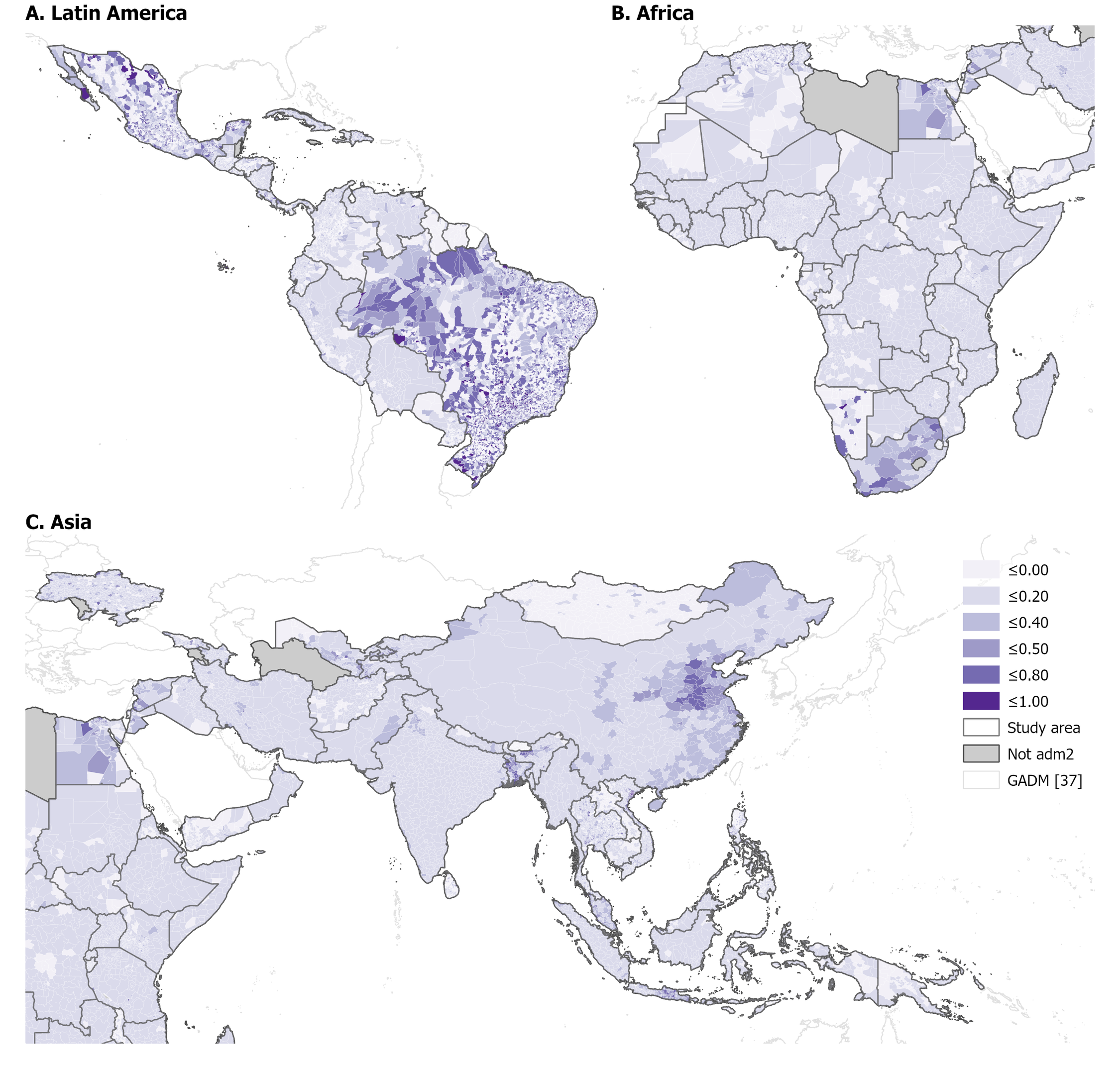

Supplement: S17 Fig — Proportion of children under 1 year of age in peri-urban areas estimated to have not received the first dose of the MCV vaccine in 2019 at administrative level 2 [31, 37] for Latin America (A), Africa (B), and Asia (C). (TIF) [file pgph.0001126.s017.tif]

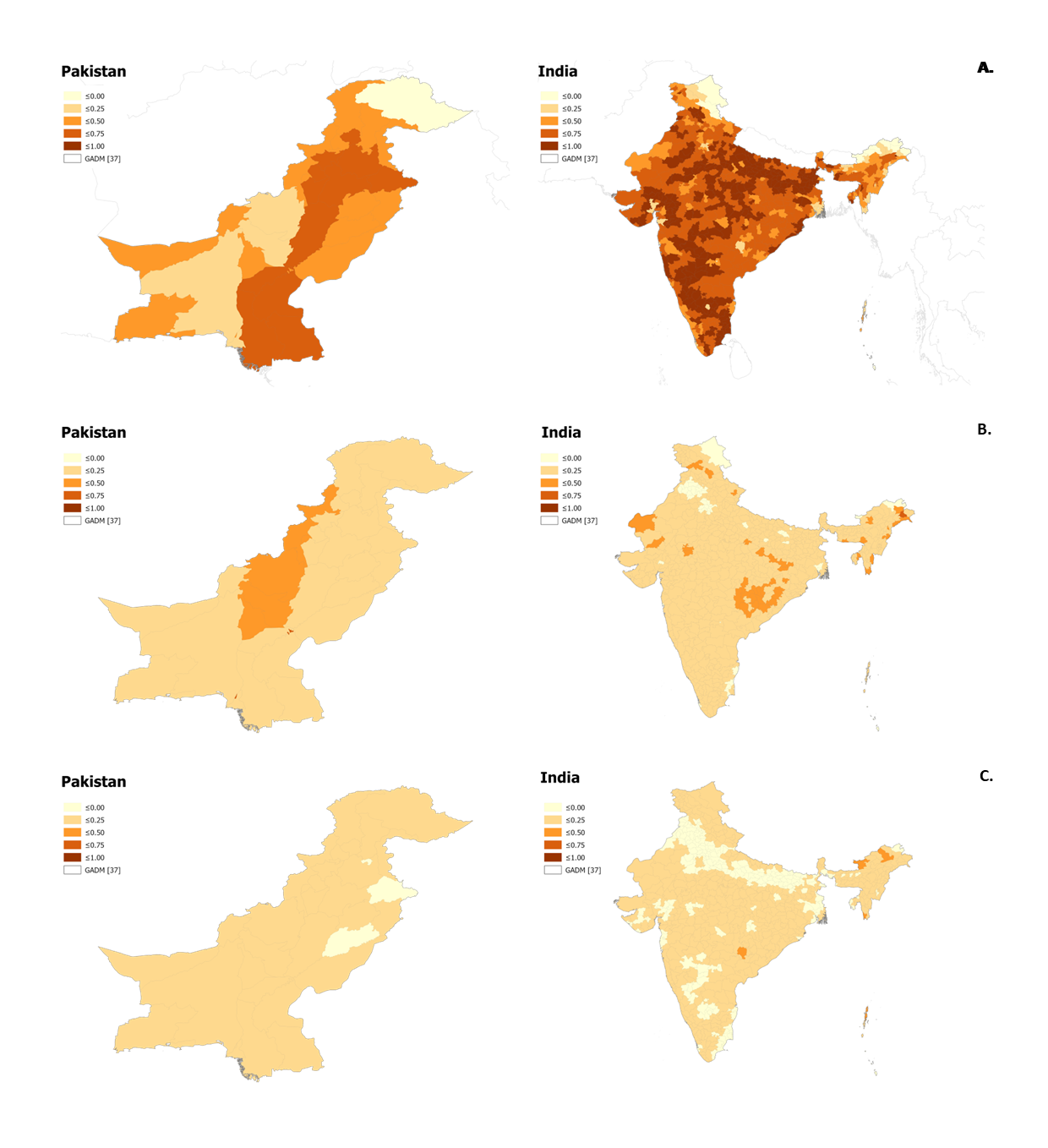

Supplement: S18 Fig — Proportion of children under 1 year of age unvaccinated for DTP1 in rural non-remote areas (Pakistan and India [31, 37]), within 0 to 60 (A), 60 to 120 (B), and 120 to 180 (C) minutes of the nearest town or city of 500,000 people or more. (TIF) [file pgph.0001126.s018.tif]

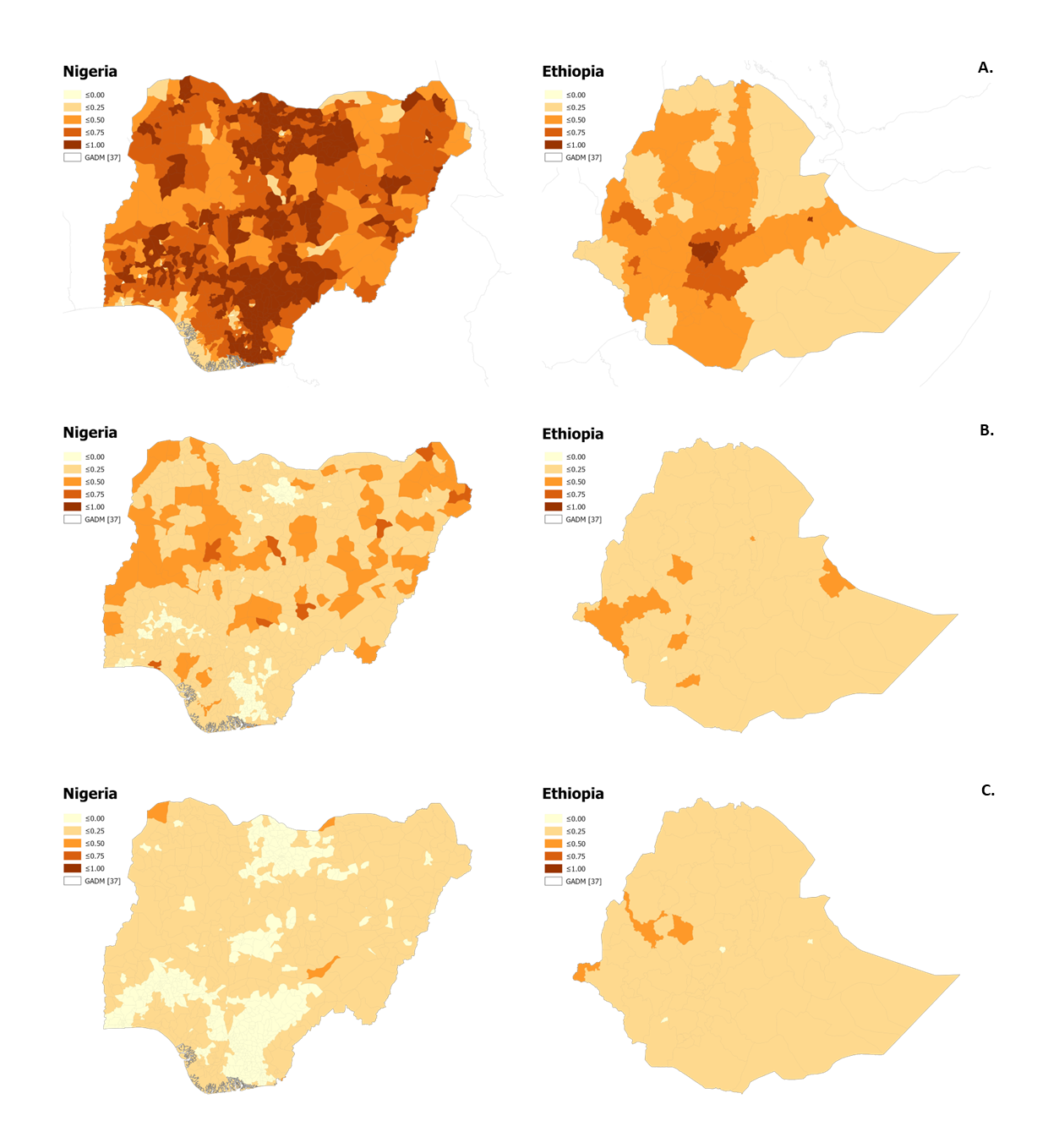

Supplement: S19 Fig — Proportion of children under 1 year of age unvaccinated for DTP1 in rural non-remote areas (Nigeria and Ethiopia [31, 37]), within 0 to 60 (A), 60 to 120 (B), and 120 to 180 (C) minutes of the nearest town or city of 500,000 people or more. (TIF) [file pgph.0001126.s019.tif]

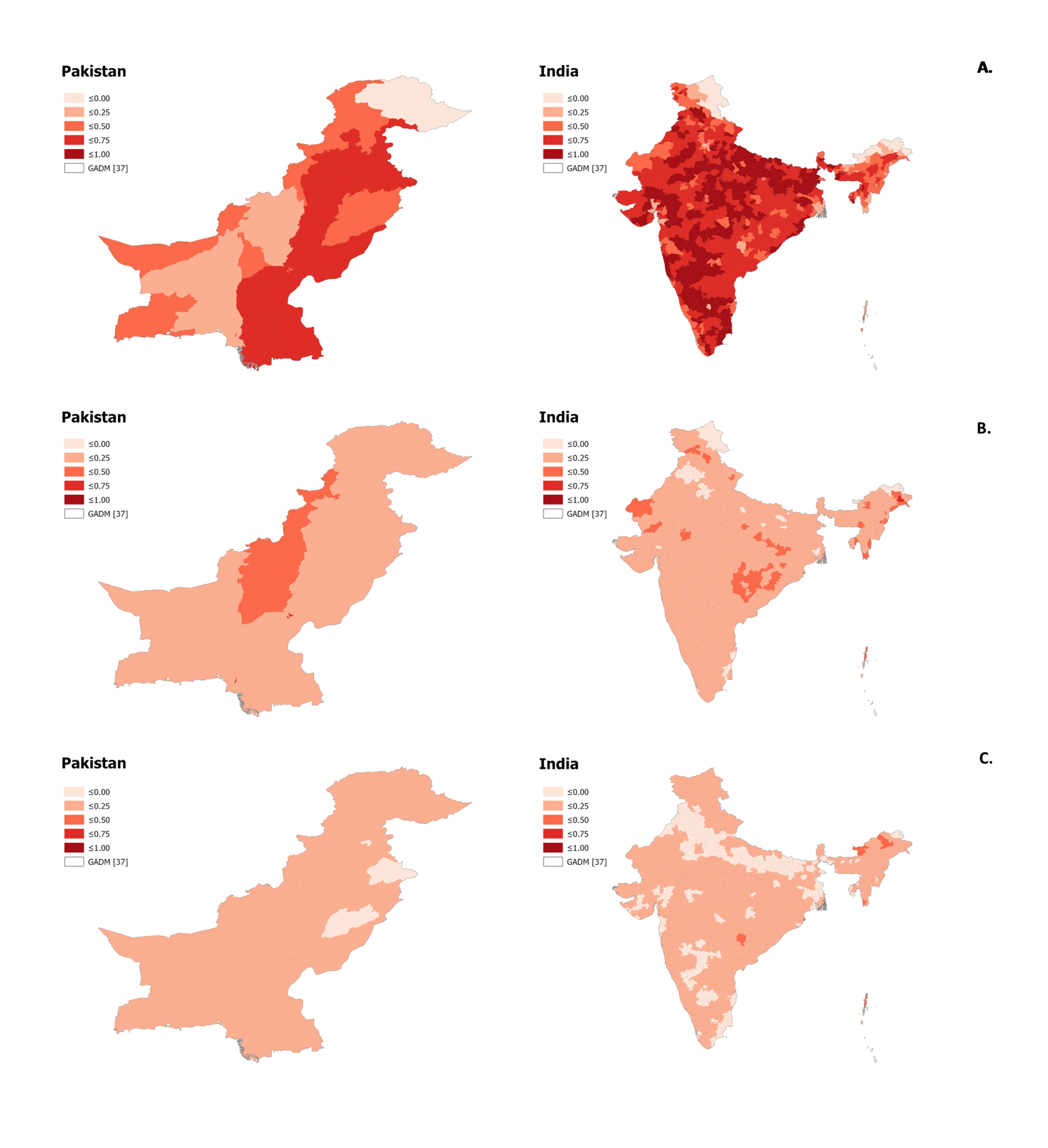

Supplement: S20 Fig — Proportion of children under 1 year of age unvaccinated for DTP3 in rural non-remote areas (Pakistan and India [31, 37]), within 0 to 60 (A), 60 to 120 (B), and 120 to 180 (C) minutes of the nearest town or city of 500,000 people or more. (TIF) [file pgph.0001126.s020.tif]

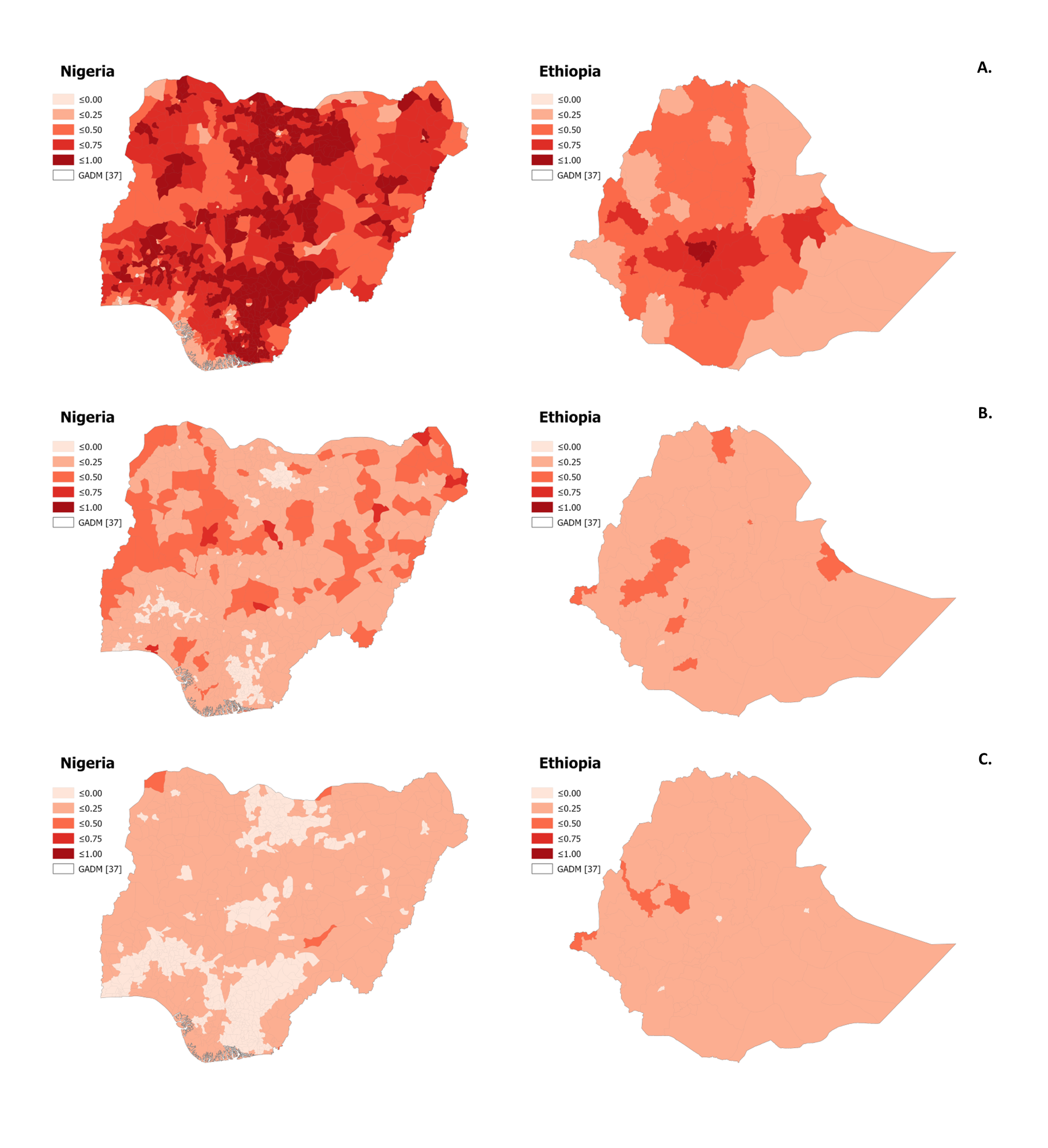

Supplement: S21 Fig — Proportion of children under 1 year of age unvaccinated for DTP3 in rural non-remote areas (Nigeria and Ethiopia [31, 37]), within 0 to 60 (A), 60 to 120 (B), and 120 to 180 (C) minutes of the nearest town or city of 500,000 people or more. (TIF) [file pgph.0001126.s021.tif]

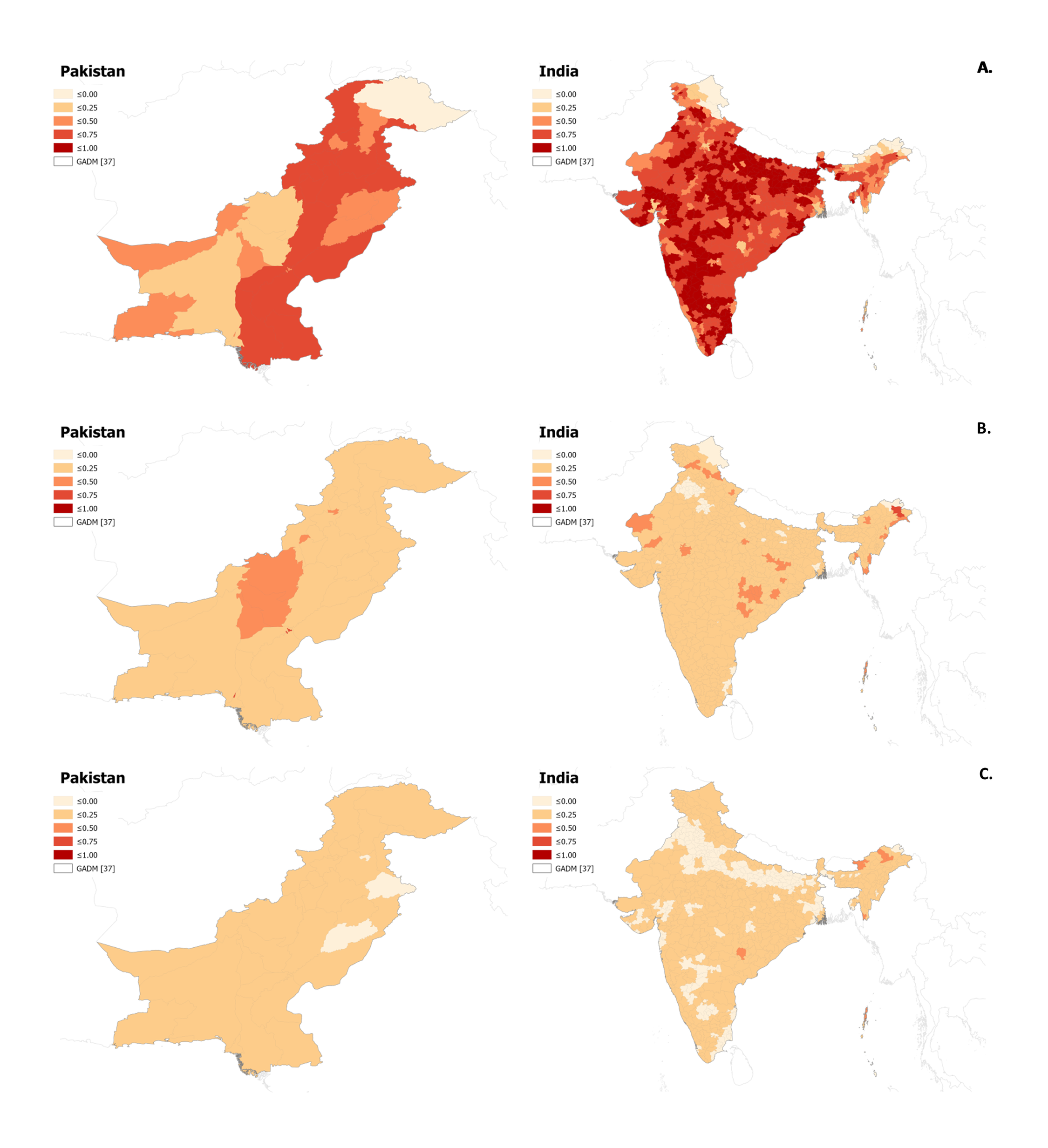

Supplement: S22 Fig — Proportion of children under 1 year of age unvaccinated for MCV1 in rural non-remote areas (Pakistan and India [31, 37]), within 0 to 60 (A), 60 to 120 (B), and 120 to 180 (C) minutes of the nearest town or city of 500,000 people or more. (TIF) [file pgph.0001126.s022.tif]

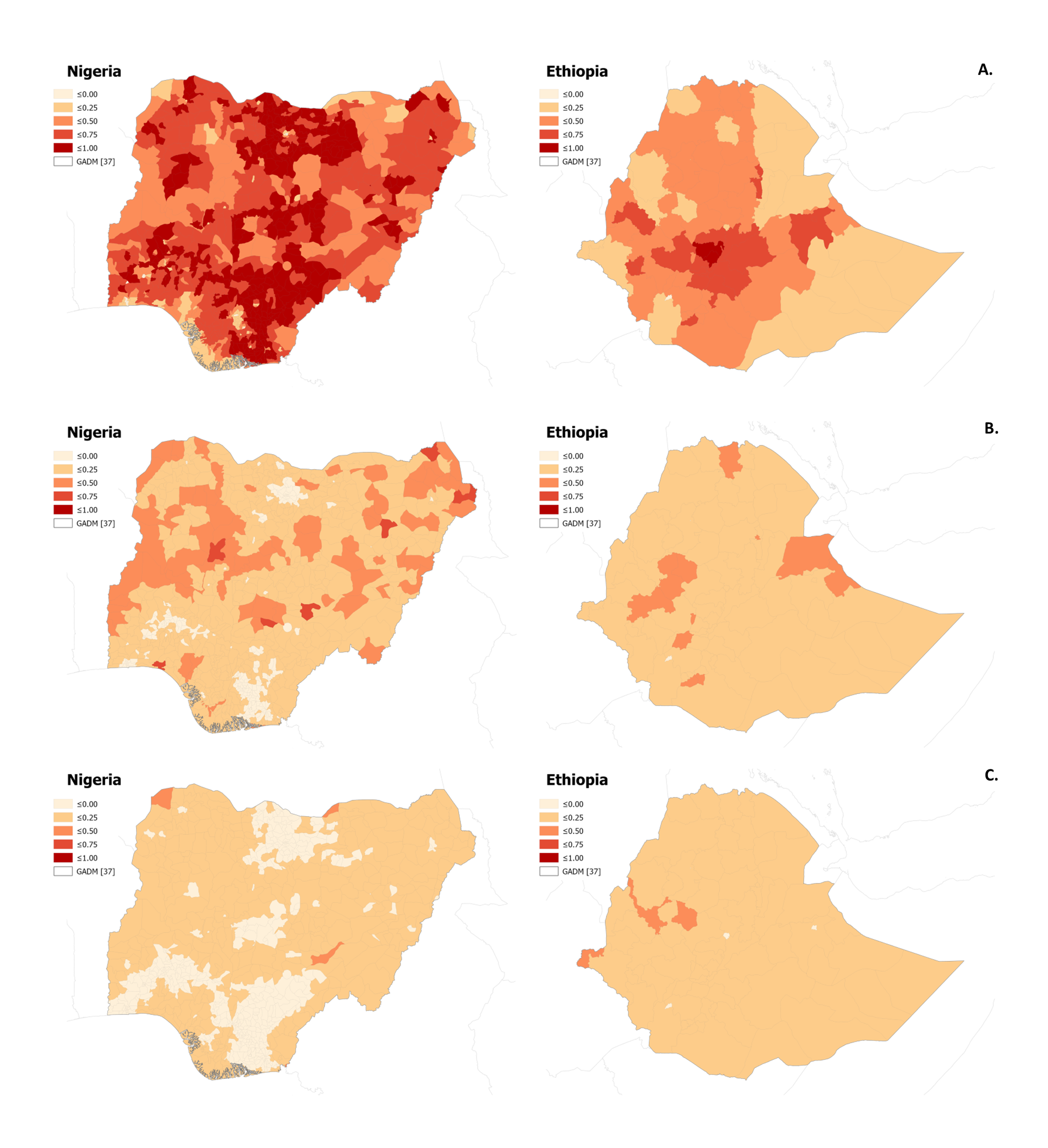

Supplement: S23 Fig — Proportion of children under 1 year of age unvaccinated for MCV1 in rural non-remote areas (Nigeria and Ethiopia [31, 37]), within 0 to 60 (A), 60 to 120 (B), and 120 to 180 (C) minutes of the nearest town or city of 500,000 people or more. (TIF) [file pgph.0001126.s023.tif]

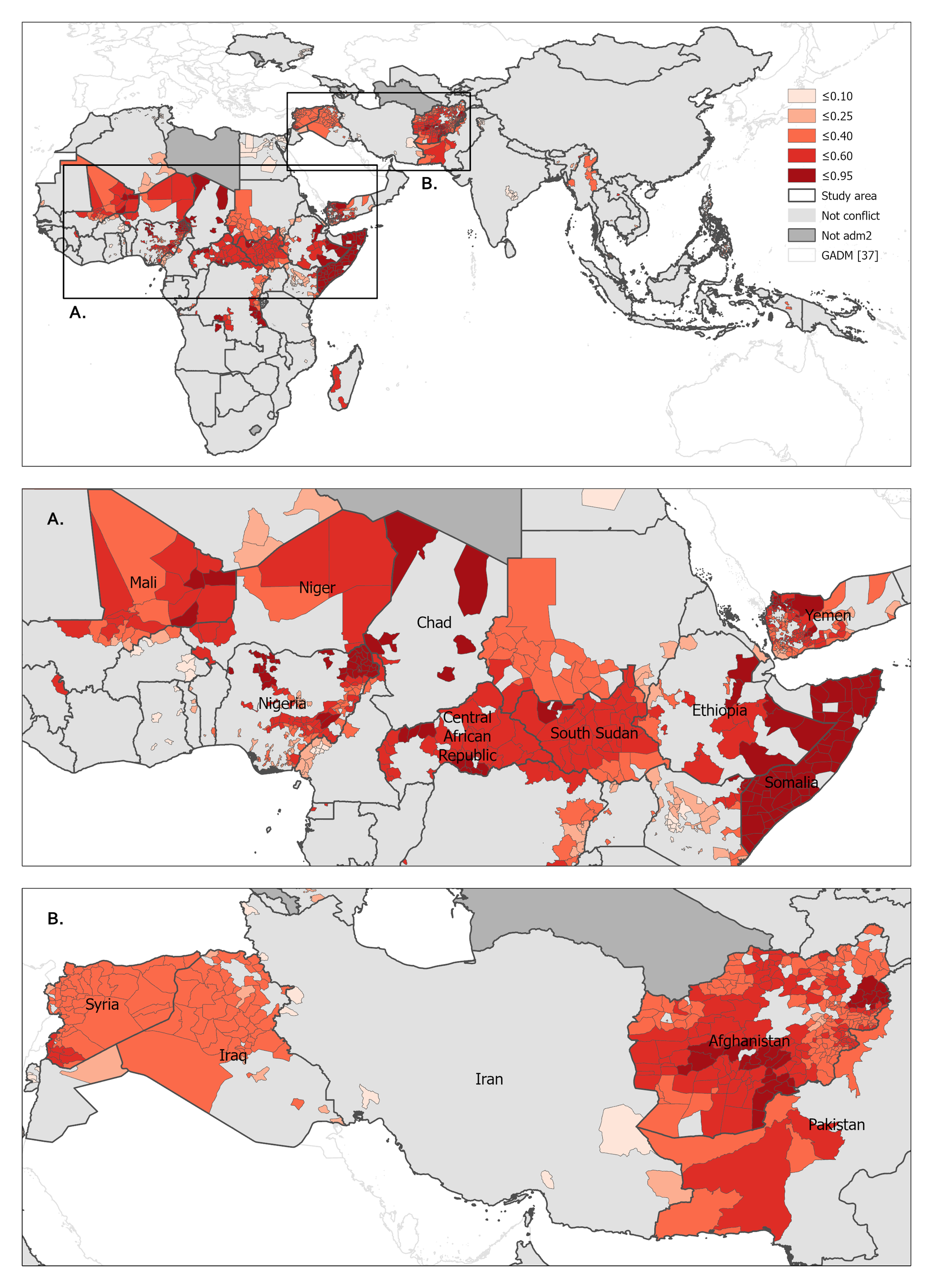

Supplement: S24 Fig — Proportion of children under 1 year of age in conflict-affected areas (broad definition) estimated to have not received the third dose of the DTP vaccine in 2019 at administrative level 2 for Africa and Asia [31, 37], with close ups shown for northern sub-Saharan Africa (A), and the Middle East/West Asia region (B). (TIF) [file pgph.0001126.s024.tif]

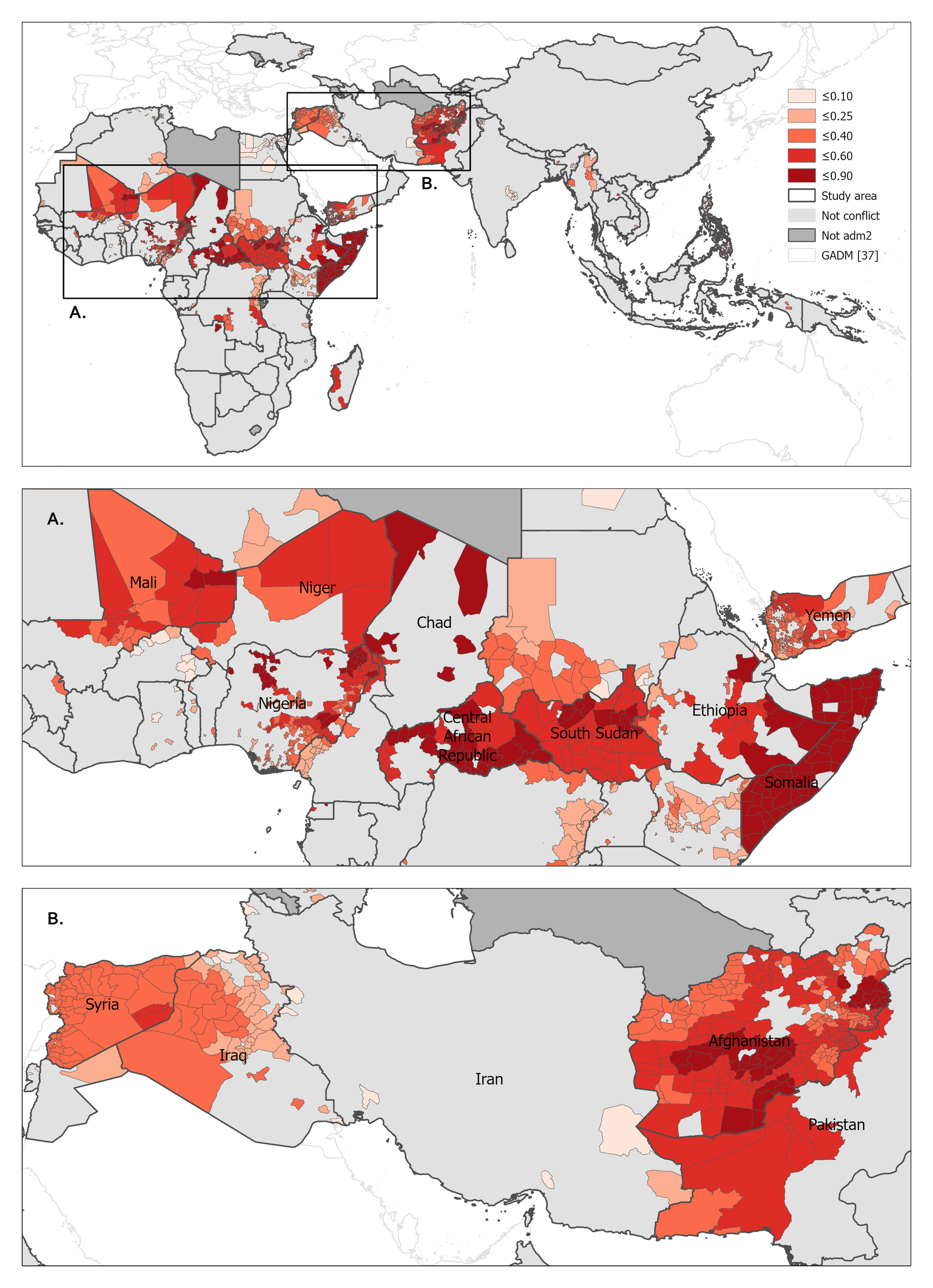

Supplement: S25 Fig — Proportion of children under 1 year of age in conflict-affected areas (broad definition) estimated to have not received the first dose of the MCV vaccine in 2019 at administrative level 2 for Africa and Asia [31, 37], with close ups shown for northern sub-Saharan Africa (A), and the Middle East/West Asia region (B). (TIF) [file pgph.0001126.s025.tif]

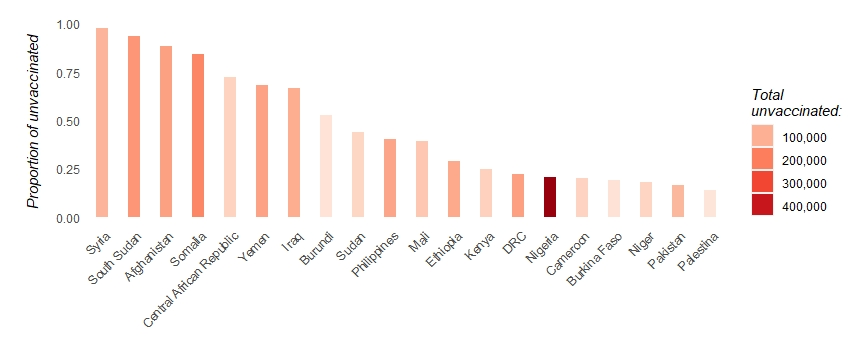

Supplement: S26 Fig — (TIF) [file pgph.0001126.s026.tif]

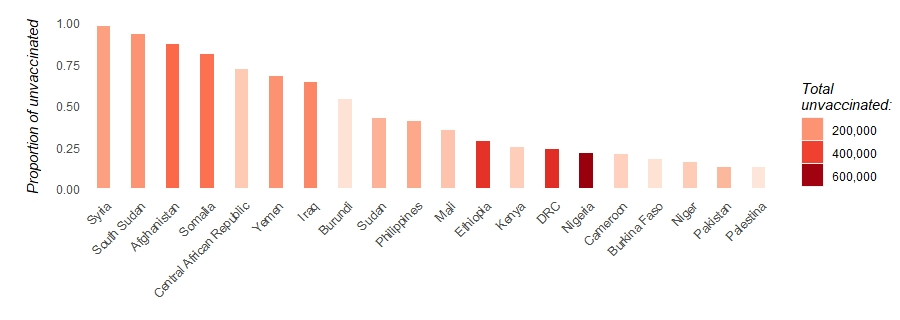

Supplement: S27 Fig — (TIF) [file pgph.0001126.s027.tif]

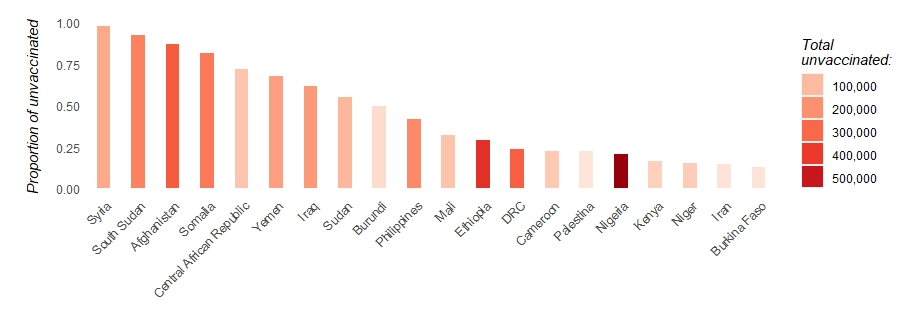

Supplement: S28 Fig — (TIF) [file pgph.0001126.s028.tif]

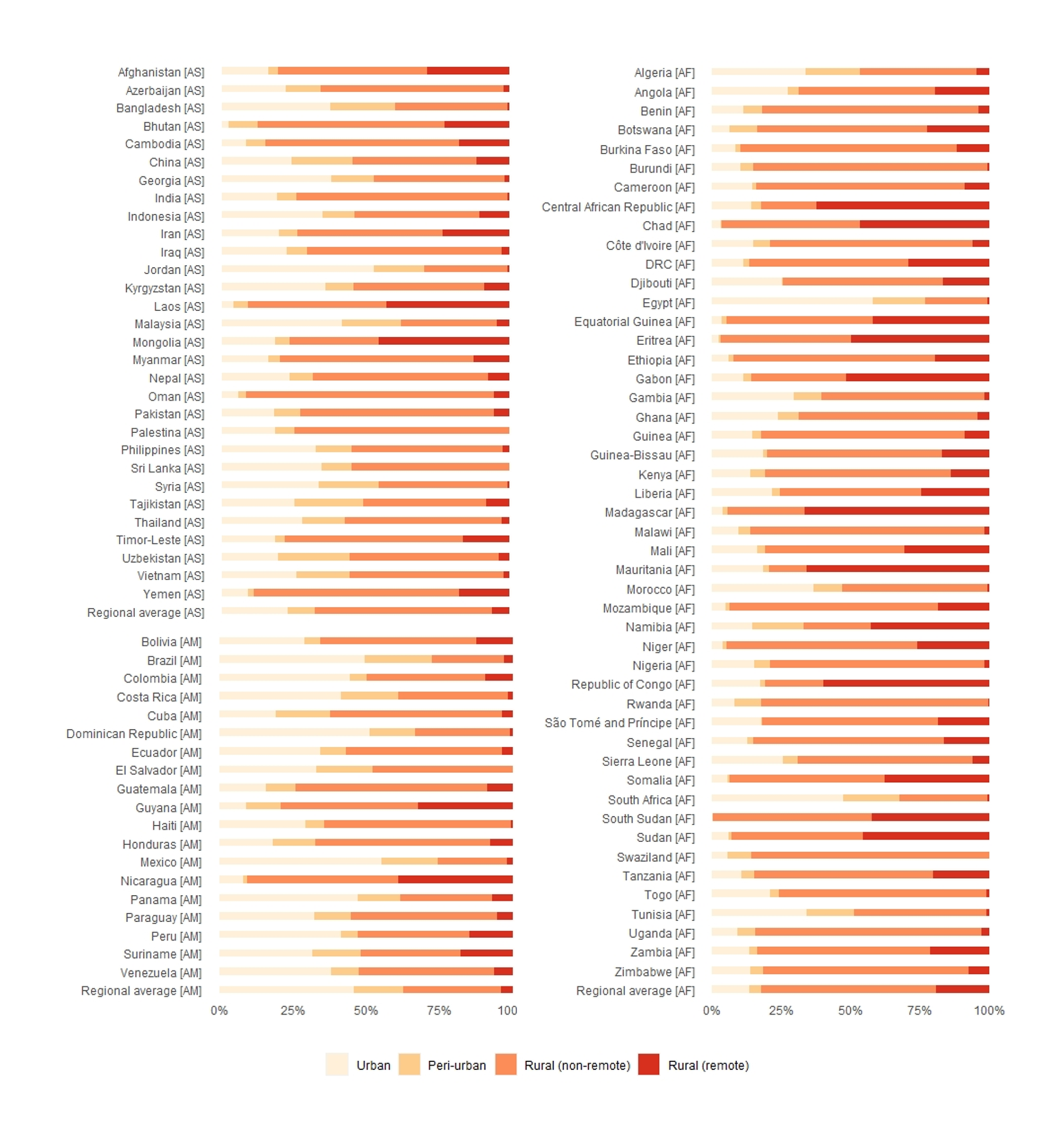

Supplement: S29 Fig — (TIF) [file pgph.0001126.s029.tif]

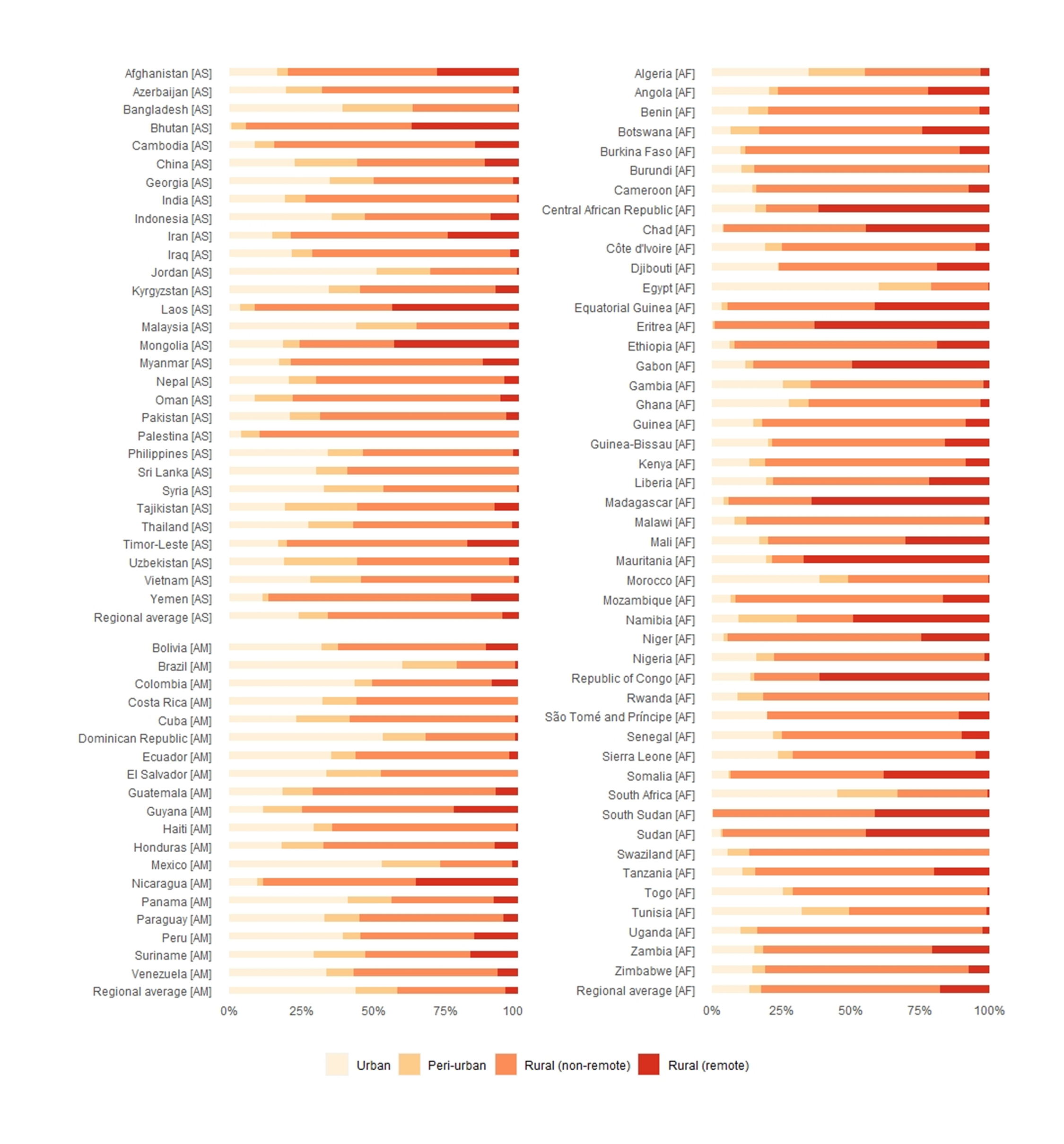

Supplement: S30 Fig — (TIF) [file pgph.0001126.s030.tif]

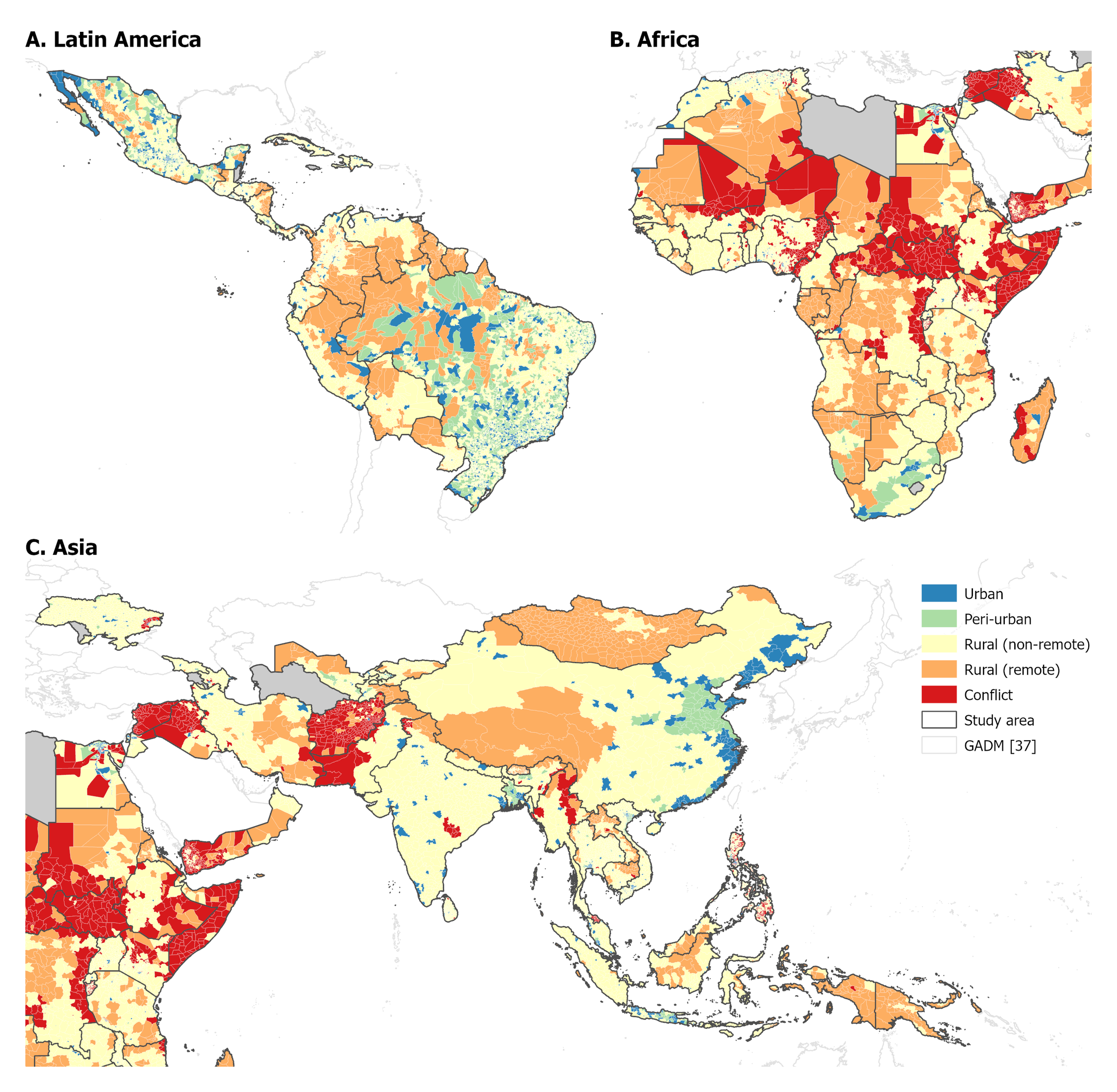

Supplement: S31 Fig — Map highlighting the geographical setting with the estimated greatest number of children under 1 years of age in 2019 not receiving DTP3 at administrative level 2 [31, 37] for Latin America (A), Africa (B), and Asia (C). (TIF) [file pgph.0001126.s031.tif]

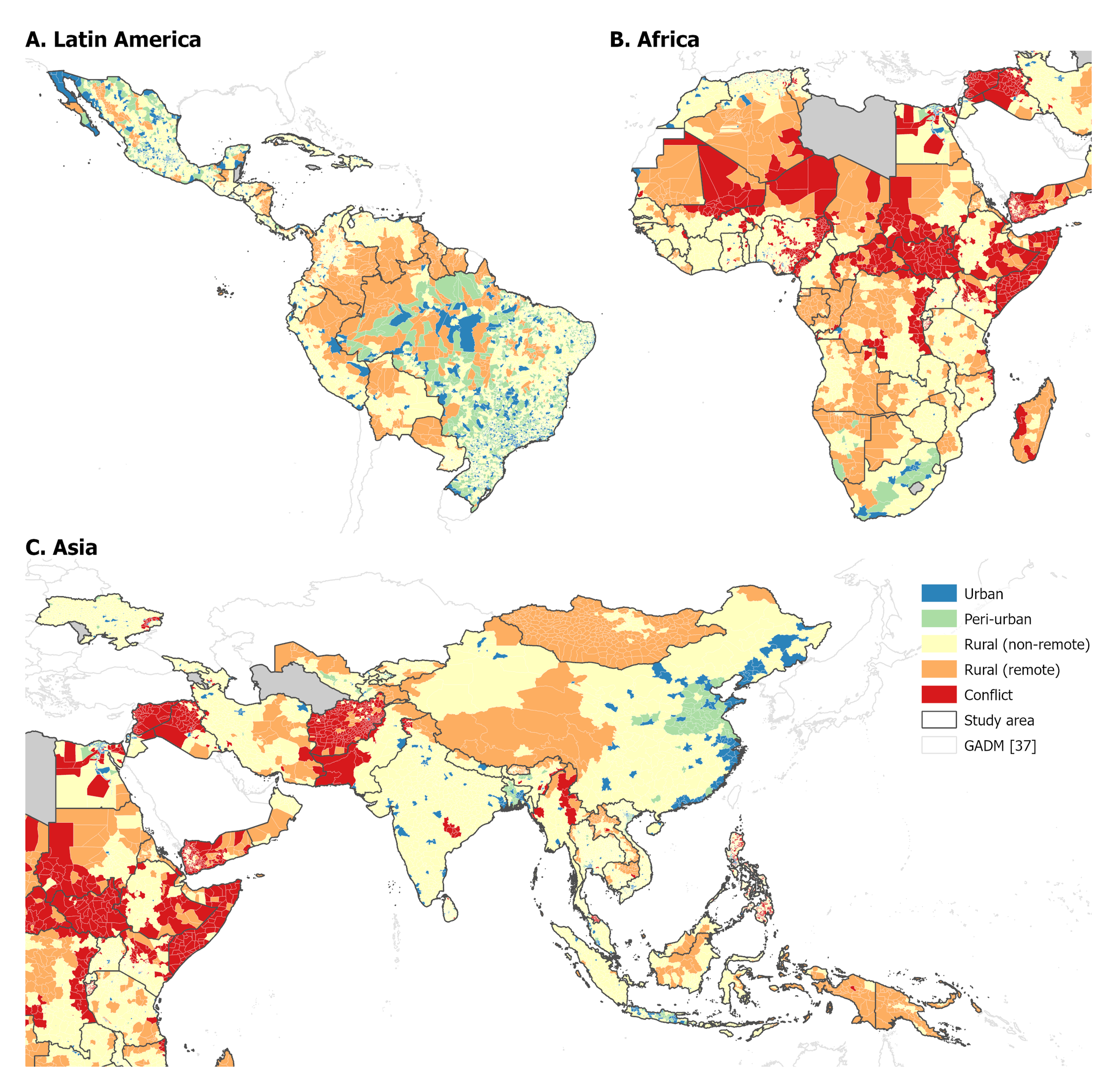

Supplement: S32 Fig — Map highlighting the geographical setting with the estimated greatest number of children under 1 years of age in 2019 not receiving MCV1 at administrative level 2 [31, 37] for Latin America (A), Africa (B), and Asia (C). (TIF) [file pgph.0001126.s032.tif]
