# Supplementary material for: Estimates of the number and distribution of zero-dose and under-immunised children across remote-rural, urban, and conflict-affected settings in low and middle-income countries
Source: PLOS Glob Public Health. 2022 Oct 26;2(10):e0001126. doi: 10.1371/journal.pgph.0001126 (PMC10021885; doi:10.1371/journal.pgph.0001126)
Supplement: Attachment — Submitted filename: Response to Reviewers.docx [file pgph.0001126.s033.docx]

8^th^ September 2022

To: Dr. Abram L. Wagner

Academic Editor, PLOS Global Public Health

Dear Dr. Abram L. Wagner:

**Subject: Submission of revised manuscript [PGPH-D-22-01112]**

Thank you for your email and for the opportunity to revise our manuscript entitled *‘Estimates of the numbers and distribution of zero-dose and under-immunised children across remote-rural, urban, and conflict-affected settings in low and middle-income countries.’* We have carefully reviewed and revised the manuscript according to the points raised by the reviewers and for the journal requirements. Please find our responses detailed below indicating how we have addressed each point and the changes that have been made.

**Journal Requirements:**

1. Please update the completed 'Competing Interests' statement. Please declare all competing interests beginning with the statement “I have read the journal's policy and the authors of this manuscript have the following competing interests:”.

- The above text has been added to the ‘Competing Interests’ statement.

2. Please amend your online detailed Financial Disclosure statement. This is published with the article. It must therefore be completed in full sentences and contain the exact wording you wish to be published.

a. State the initials, alongside each funding source, of each author to receive each grant.
b. State what role the funders took in the study. If the funders had no role in your study, please state: “The funders had no role in study design, data collection and analysis, decision to publish, or preparation of the manuscript.”

- The financial Disclosure statement has been revised to a) include the initials of each author to receive the grant, and b) the above text added to state that the funders had no role in the study.

3. Please provide separate figure files in .tif or .eps format only and ensure that all files are under our size limit of 10MB.

- All figures have been converted to .tif format and are under the 10MB size limit, and all figures have been uploaded to PACE to ensure that they meet PLOS guidelines.

4. We have noticed that you have a list of Supporting Information legends in your manuscript. However, there are no corresponding files uploaded to the submission. Please upload them as separate files with the item type 'Supporting Information'.

- All Supporting information files have been uploaded as a compressed zip file named ‘Supporting Information’.

5. All figures and supporting information files will be published under the Creative Commons Attribution License (creativecommons.org/licenses/by/4.0/). Authors retain ownership of the copyright for their article and are responsible for third-party content used in the article.

Figures 1, 2, 3, and 5: please (a) provide a direct link to the base layer of the map used and ensure this is also included in the figure legend; (b) provide a link to the terms of use / license information for the base layer. We cannot publish proprietary or copyrighted maps (e.g. Google Maps, Mapquest) and the terms of use for your map base layer must be compatible with our CC-BY 4.0 license.

- The base layer used for the maps is compatible with the CC-BY 4.0 license and we have (a) provided a reference to the data used in the methods section, the map figure captions and map legends and (b) provided a reference to the terms of use/licence information to the base layer in each of the map figure captions.

6. Please review your reference list to ensure that it is complete and correct. If you have cited papers that have been retracted, please include the rationale for doing so in the manuscript text, or remove these references and replace them with relevant current references. Any changes to the reference list should be mentioned in the rebuttal letter that accompanies your revised manuscript. If you need to cite a retracted article, indicate the article’s retracted status in the References list and also include a citation and full reference for the retraction notice.

- The reference list has been reviewed to ensure that it is correct and complete.

**Review Comments to the Author:**

**Reviewer #2:** Thank you, authors, for the opportunity to review your work. The work is very interesting and adds detailed input to existing knowledge. I just have a few concerns highlighted below;

Line 25 …….estimate…. replace with ….estimated….
Line 28 ….. we find… replace with ………we found…..
Line 29 delete We estimate…. It should read as Of the…….
Lines 57-61 is just one sentence consider breaking it into 2 or 3 sentences

- The suggested changes have been made to the text.

Overall, the article was well written. I would just advise the authors to revise some of the long sentences and make them shorter as it is easier to engage with shorter sentences than long sentences. Well done.

**Reviewer #3:** Abstract
Redesign the entire abstract following the IMRaD structure. Start from the general to the specific in the introduction. The methodology should also be included in the summary. Clearly state your results (rephrasing). The conclusion must be well separated and must support the results of the work.

- The abstract text has been revised and reformatted to incorporate the above comments.

Results
Put the titles of the tables at the top
Remove the percentage symbol inside table 1 by adopting for example n(%). This form of presentation is easy to understand. In the header of table 1, put for example DTP1 (N=14,030,486) and so on for the other variables in the header.

- The above changes have been made to the table.

Review the references. I noticed that the numbers are missing. Also review the pages.
For example, on line 497 for reference 33, it is ............26(12):1-4.

- The references have been reviewed and the page numbers and as well as any missing numbers completed. Some duplicate references have been removed.

**Reviewer #4:** Title:
Remove one 'and' and just use comma. Hence the title should be:

*'Estimates of the number, distribution of zero-dose and under-immunised children across remote-rural, urban, and conflict-affected settings in low and middle-income countries’*

- We have considered the suggested revised title. We believe that the ‘and’ is needed between ‘numbers’ and ‘distribution’ for it to reflect the nature of the analysis. The wording of ‘numbers’ has been revised to ‘number’ as suggested.

Methods and results:
only Univariate level i.e,. proportions have been calculated. Can you go farther to the level of bivariate and multivariate levels ie. to find out Associations of the factors?

- Further analysis has not been carried out as the focus of the analysis was to look at the numbers and distributions of un- and under-vaccinated children globally at the univariate level. Analysis has been carried out looking at the multivariate factors that are associated with un- and under-vaccination in a complementary paper of ours, which we cite and alert readers to in the manuscript: <https://doi.org/10.1371/journal.pgph.0000244>.

**Additional Comments:**

Lines 48 – 50, page 3; “In 2019, 13.6 million children failed to receive even a first dose … rising significantly to 17.1 million in 2020…” The authors may want to update this by including the recent WHO coverage estimates reflecting the sustained decline in DTP3 coverage in 2021 as well.

- We have added additional text to include a reference to the recent WHO coverage estimates highlighting a sustained decline in DTP3 coverage in 2021.

Line 132, page 6; “…estimates of the numbers of unvaccinated PU1 living in urban slums in this analysis.” The authors should provide the full term for “PU1” unless they are still referring to “U1” in which case this should be corrected for clarity.

- This text has been revised to ‘U1’ instead of ‘PU1’.

Line 257, page 13; “…regions where large proportions of U1 were unvaccinated, including much of Somalia…” As part of this sentence, kindly provide the proportions referred to for ease of reference.

- The proportions have been added to the text.

Lines 286 – 288, page 14; “For example, in India, the … However, in Africa, a greater proportion…” Given that the authors appear to be comparing countries in the different would regions, I suggest the following edits for ease of clarity: “For example, in a South Asian country like India, the … However, within Africa …”

- We have revised the text to the above suggestion.

Line 290, page 14; “…remote (non-rural) and remote (rural) areas, however in Brazil, higher numbers…” The authors should clarify if this classification differs from the one used throughout the manuscript, i.e., “rural (non-remote) and rural (remote)”.

- Yes, this is the same definition – this has been revised.

Lines 349 – 350, page 16; “…the choice of threshold or data to use naturally effects the estimates produced…” Do the authors mean “affects”?

- Yes, this text has been revised.

Lines 391 – 392, page 18; “…and for the need more country-specific and targeted approaches to immunisation so…” Kindly rephrase this sentence for better clarity.

- This sentence has been revised for better clarity.

We hope that the revised version is now suitable for publication and look forward to hearing from you.

Yours Sincerely,

Adelle Wigley (on behalf of all authors)
